# Schistosome egg antigens, including the glycoprotein IPSE/alpha-1, trigger the development of regulatory B cells

**DOI:** 10.1371/journal.ppat.1006539

**Published:** 2017-07-28

**Authors:** Simone Haeberlein, Katja Obieglo, Arifa Ozir-Fazalalikhan, Mathilde A. M. Chayé, Henrike Veninga, Luciën E. P. M. van der Vlugt, Astrid Voskamp, Louis Boon, Joke M. M. den Haan, Lotte B. Westerhof, Ruud H. P. Wilbers, Arjen Schots, Gabriele Schramm, Cornelis H. Hokke, Hermelijn H. Smits

**Affiliations:** 1 Department of Parasitology, Leiden University Medical Center, Leiden, Netherlands; 2 Department of Molecular Cell Biology and Immunology, VU University Medical Center, Amsterdam, Netherlands; 3 Bioceros, Utrecht, Netherlands; 4 Plant Science Department, Wageningen University and Research Centre, Droevendaalsesteeg, Wageningen, Netherlands; 5 Experimental Pneumology, Priority Research Area Asthma & Allergy, Research Center Borstel, Parkallee, Borstel, Germany; Uniformed Services University of the Health Sciences, UNITED STATES

## Abstract

Infection with the helminth *Schistosoma (S*.*) mansoni* drives the development of interleukin (IL)-10-producing regulatory B (Breg) cells in mice and man, which have the capacity to reduce experimental allergic airway inflammation and are thus of high therapeutic interest. However, both the involved antigen and cellular mechanisms that drive Breg cell development remain to be elucidated. Therefore, we investigated whether *S*. *mansoni* soluble egg antigens (SEA) directly interact with B cells to enhance their regulatory potential, or act indirectly on B cells via SEA-modulated macrophage subsets. Intraperitoneal injections of *S*. *mansoni* eggs or SEA significantly upregulated IL-10 and CD86 expression by marginal zone B cells. Both B cells as well as macrophages of the splenic marginal zone efficiently bound SEA *in vivo*, but macrophages were dispensable for Breg cell induction as shown by macrophage depletion with clodronate liposomes. SEA was internalized into acidic cell compartments of B cells and induced a 3-fold increase of IL-10, which was dependent on endosomal acidification and was further enhanced by CD40 ligation. IPSE/alpha-1, one of the major antigens in SEA, was also capable of inducing IL-10 in naïve B cells, which was reproduced by tobacco plant-derived recombinant IPSE. Other major schistosomal antigens, omega-1 and kappa-5, had no effect. SEA depleted of IPSE/alpha-1 was still able to induce Breg cells indicating that SEA contains more Breg cell-inducing components. Importantly, SEA- and IPSE-induced Breg cells triggered regulatory T cell development *in vitro*. SEA and recombinant IPSE/alpha-1 also induced IL-10 production in human CD1d^+^ B cells. In conclusion, the mechanism of *S*. *mansoni*-induced Breg cell development involves a direct targeting of B cells by SEA components such as the secretory glycoprotein IPSE/alpha-1.

## Author summary

Infection with helminth parasites is known to be inversely associated with hyper-inflammatory disorders. While *Schistosoma (S*.*) mansoni* has been described to exert its down-modulatory effects on inflammation by inducing a network of regulatory immune cells such as regulatory B (Breg), the mechanisms of Breg cell induction remain unclear. Here, we use *in vivo* and *in vitro* approaches to show that antigens from *S*. *mansoni* eggs, among which the major glycoprotein IPSE/alpha-1, directly interact with splenic marginal zone B cells of mice which triggers them to produce the anti-inflammatory cytokine IL-10 and their capacity to induce regulatory T (Treg) cells. We also found that IPSE/alpha-1 induces IL-10 in human CD1d^+^ B cells, and that both natural and recombinant IPSE/alpha-1 are equally effective in driving murine and human Breg cells. Our study thus provides insight into the mechanisms of Breg cell induction by schistosomes, and an important step towards the development of helminth-based treatment strategies against hyper-inflammatory diseases.

## Introduction

Helminths can persist for up to decades in the human host. This is hypothesized to be, at least in part, because of their evolutionarily adapted relationship with the host [1]. Helminths are well-known for their strong capacity to promote the regulatory arm of the hosts immune system, thereby prolonging their survival within the host [2]. As a bystander effect, helminths can also suppress immune responses to other antigens, such as allergens and auto-antigens, and other pathogens. This bystander effect seems to be so pronounced that it may prevent the development of inflammatory diseases. Indeed, both epidemiological studies and mouse models show a clear protective role of helminths against various forms of auto-immunity, allergic airway inflammation, colitis etc. [3,4,5,6,7]. The formation of a network of regulatory immune cells plays a crucial role for the protective effect. Helminth infection, and in particular infection with schistosomes such as *Schistosoma (S*.*) mansoni* are well-known to induce regulatory B (Breg) cells [8–15], a relatively new member in the network of regulatory immune cells. Breg cells have gained considerable attention due to their ability to down-modulate inflammation in a variety of conditions ranging from autoimmune disorders such as experimental autoimmune encephalomyelitis (EAE), collagen-induced arthritis (CIA), lupus and chronic colitis to anaphylactic and allergic airway inflammation [8,10–12,16–23]. Regulatory B cells suppress pro-inflammatory immune responses via several mechanisms, of which the ones best described are the expression of the regulatory cytokine interleukin-10 (IL-10) and induction of regulatory T (Treg) cells [24].

We previously reported the induction of Breg cells by schistosome infection in both mouse and human, and found the most potent IL-10-producing Breg cells within the human CD1d^+^ B cell subset. This corresponds to the CD1d^+^CD23^low^CD21^+^ marginal zone (MZ) B cell subset in mice, which efficiently reduced experimental allergic airway inflammation in our model [12]. The cellular mechanisms to achieve Breg cell induction as well as the nature of the B cell-activating *S*. *mansoni* antigens however remain largely unknown. The identification of relevant stimulatory molecules and optimal Breg cell-inducing conditions is a critical step in enhancing the activity of Breg cells for use as a new therapeutic tool against inflammatory diseases.

Both an indirect induction of a regulatory phenotype in B cells by activation of accessory cell types, as well as a direct binding and interaction between *S*. *mansoni* antigens and B cells via pattern recognition receptors (PRRs) such as Toll-like receptors (TLRs) expressed on B cells [25] are plausible options. In the splenic MZ, located at the border of white and red pulp, MZ B cells are nested between SIGN-R1^+^ MZ macrophages and Siglec-1^+^ metallophilic macrophages [26]. MZ macrophages not only fulfill a main function in sensing blood-borne pathogens, but also perform functional interactions with MZ B cells. These interactions have important implications for the maintenance of the MZ itself and the function of MZ B cells and macrophages [27–29]. Hence, it is therefore tempting to speculate that MZ macrophages are a prime candidate as Breg cell induction partner. On the other hand, the direct ligation of various TLRs on B cells, including TLR2, TLR4, TLR7 and TLR9, has been described to induce IL-10 production [30,31]. In addition, BCR and CD40 engagement were described to be involved in IL-10-dependent regulatory B cell function in models of EAE, CIA, and contact hypersensitivity [17–19,32].

In the current study, we tested the hypothesis that eggs and/or egg-derived excretory-secretory molecules from *S*. *mansoni*, without the context of natural infection, are sufficient to drive Breg cell development by activating splenic MZ B cells. In addition, we investigated whether Breg cells are induced indirectly by activation of accessory cell types in the MZ, or by direct binding and interaction via PRRs on B cells. We found that egg antigens drive Breg cell development *in vivo* and *in vitro* by direct interaction with splenic B cells, which after binding and internalization of egg antigens secrete elevated levels of IL-10 and are capable of driving Treg cell development. The egg antigen-induced Breg cell development was independent of macrophages of the marginal zone but was enhanced by CD40 ligation. Most importantly, we identified the egg glycoprotein IPSE/alpha-1 as a single molecule from *S*. *mansoni* that is capable to induce Breg cells both in mice and man. This knowledge will assist to further define helminth-specific conditions for the generation of Breg cells to be used in therapeutic approaches.

## Results

### Schistosome egg antigens drive the development of Breg cells *in vivo*

**Fig 1:**
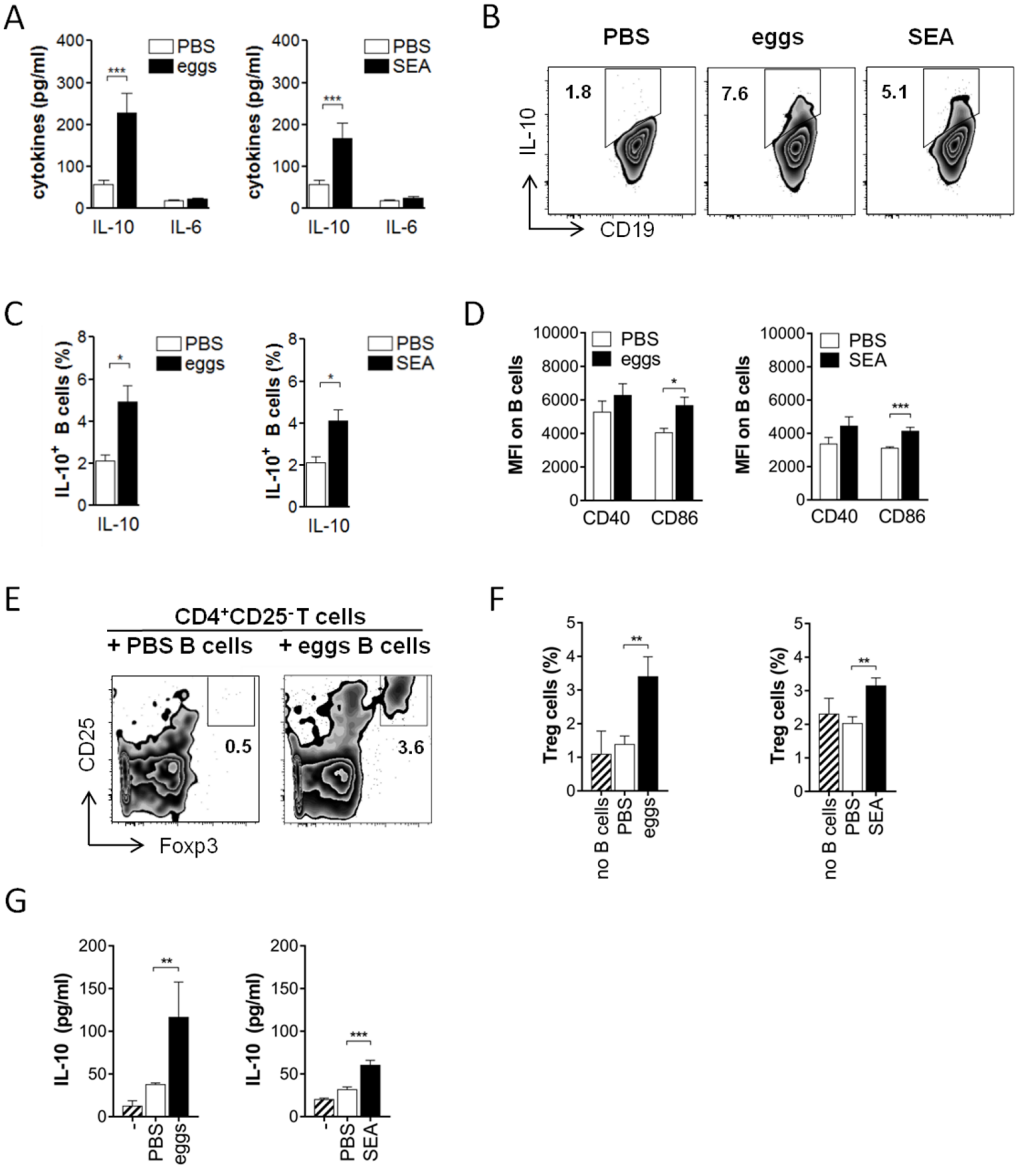
Schistosome eggs and SEA (soluble egg antigen) drive development of Breg cells *in vivo*. C57BL/6 mice were i.p. injected with two doses of 5000 *S*. *mansoni* eggs or 100 μg SEA in PBS, or PBS as control. At day 14, CD19^+^ MACS-isolated splenic B cells were restimulated with SEA (20 μg/ml) for 2 days. **(A)** Cytokine concentration in culture supernatants as determined by ELISA. **(B, C)** Representative FACS plots (B) and summary (C) for intracellular IL-10 expression of B cells after addition of Brefeldin A to the last 4 hours of the culture. **(D)** Mean fluorescence intensity of CD40 and CD86 expression. **(E-G)** SEA-restimulated B cells were co-cultured for 4 days with CD25-depleted CD4 T cells. Frequency of CD25^+^Foxp3^+^ Treg cells after co-culture in representative FACS plots **(E)** and summary **(F)** is shown. **(G)** IL-10 concentration in culture supernatants after co-culture. Summary of 4 experiments with N = 12–16 (A-D) or 2 experiments with N = 8–9 (E-G). Significant differences by Mann-Whitney test are indicated with * p < 0.05, ** p < 0.01, *** p < 0.001.

To elucidate the mechanism by which *S*. *mansoni* can drive the development of Breg cells, we first investigated whether schistosome eggs or their soluble antigens were sufficient to drive Breg cell development *in vivo*, without the context of natural infection. Intraperitoneal treatment of C57BL/6 mice with two doses of 5000 *S*. *mansoni* eggs or 100 μg of soluble egg antigens (SEA) one week apart efficiently induced IL-10 protein expression in splenic CD19^+^ B cells one week after the last injection. IL-10 protein secreted during 2 days *ex vivo* restimulation with SEA was 3 to 4-fold increased compared to the amount secreted by restimulated B cells from control-treated animals (Fig 1A), while IL-6 was unchanged. This indicates a typical cytokine expression pattern characteristic for Breg cells. The frequencies of B cells expressing intracellular IL-10 protein were likewise significantly 2-fold increased (Fig 1B and 1C). Also the surface activation marker CD86, often upregulated on activated B cells and Breg cells [33–35], was increased on splenic B cells by egg or SEA treatment, while CD40 expression was not significantly changed (Fig 1D). To verify that the observed effects are specific and exclude a general influence of protein solutions on B cell IL-10 production and activation, we treated mice with human serum albumin (HSA) as infection-unrelated control protein. We did not observe an increased IL-10 secretion (S1A Fig) or CD86 expression (S1B Fig) by B cells compared to the PBS group, and concluded that PBS is a suitable control for subsequent experiments. Furthermore, injection of eggs or egg antigens was as efficient in increasing the frequency of IL-10-expressing B cells as was chronic infection with *S*. *mansoni* (S1C Fig), and egg-injected mice continued to have an elevated B cell IL-10 production until at least 4 weeks after the last egg injection (S1D Fig), indicating that this phenotype is persisting over longer periods. SEA was purified from liver eggs and can contain LPS to variable extent. Since the TLR4 ligand LPS is known for its capacity to drive B cell IL-10 expression and Breg cell development [33,36,37], it is crucial to exclude that the Breg driving capacity by SEA *in vivo* was due to LPS contamination of the schistosome antigen preparations. Therefore, the same experiment was repeated in TLR4-deficient animals and compared to wild-type. Upon SEA treatment, IL-10 secretion of B cells as well as intracellular IL-10 and surface CD86 expression was overall comparable in both groups (S2 Fig), indicating that the Breg-inducing capacity by SEA was largely not attributable to a putative LPS contamination. To confirm the regulatory function, splenic B cells from the various groups were tested for their capacity to drive Treg cell development, an acquired phenotype previously described for splenic B cells during natural schistosome infections [12]. Indeed, splenic B cells from egg- or SEA-injected, but not control-treated, mice induced the development of CD25^+^Foxp3^+^ Treg cells during 4 day co-culture with CD25-depleted CD4 T cells (Fig 1E and 1F), which confirms the regulatory capacity of egg antigen-activated B cells *ex vivo*. As expected, IL-10 protein concentration in co-culture supernatants was only increased in presence of egg antigen-activated but not control B cells (Fig 1G). Collectively, these data show that schistosome eggs or their soluble antigens on their own are sufficient to induce IL-10-producing Breg cells *in vivo*, without the context of natural infection, and that these B cells are bona fide Breg cells that can drive Treg cell development.

### Schistosome antigens activate marginal zone B cells in the spleen

**Fig 2:**
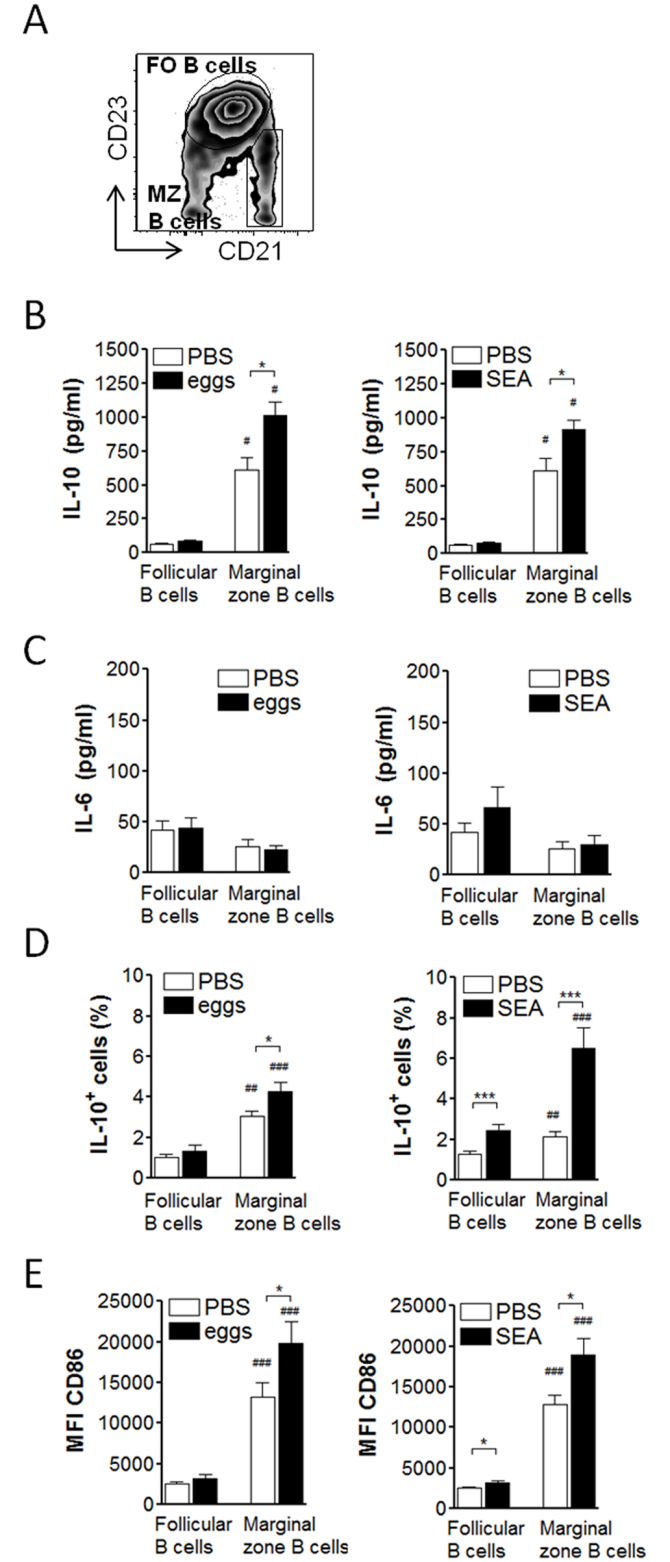
Schistosome antigens mainly activate the marginal zone B cell subset of the spleen. C57BL/6 were treated as in Fig 1, splenic follicular (FO) B cells (CD19^+^CD23^+^CD21^low^) and marginal zone (MZ) B cells (CD19^+^CD23^-^CD21^hi^) FACS sorted, and restimulated for 2 days with SEA (20 μg/ml). **(A)** Gating scheme for both B cell subsets within the CD19^+^ gated B cell population. **(B, C)** Cytokine concentration in culture supernatants as determined by ELISA. **(D)** Intracellular IL-10 expression of B cell subsets after addition of Brefeldin A to the last 4 hours of the culture. **(E)** Mean fluorescence intensity of CD86 expression of B cell subsets. Summary of 2 experiments with N = 6 (B, C) or 3–5 experiments with N = 13–21 (D, E). Significant differences by Mann-Whitney test are indicated with * p < 0.05, *** p < 0.001. Significant differences between B cell subsets by Wilcoxon paired test are indicated with ^#^ p < 0.05, ^##^ p < 0.01, ^###^ p < 0.001.

Different B cell subsets have been described to give rise to Breg cells, especially in spleen where subsets differ e.g. in tissue localization and pathogen recognition receptor expression [25,38]. We and others had previously identified CD23^low^CD21^+^ marginal zone B cells as the major IL-10-producing splenic B cell subset during chronic *Schistosoma* infection and mediating protection in a mouse model of airway inflammation [8,12]. To test whether soluble egg antigens act on the same splenic subset, we sorted splenic CD23^low^CD21^+^ marginal zone B cells from egg-, SEA-treated, or control mice for subsequent *ex vivo* restimulation and cytokine analysis, and compared this with the major splenic B cell subset, CD23^hi^CD21^-^ follicular B cells (Fig 2A). Only marginal zone B cells but not follicular B cells showed significantly increased IL-10 secretion as was measured in culture supernatants after 2 day restimulation with SEA (Fig 2B). As for total B cells (Fig 1A), also for the individual subsets IL-6 expression was not increased (Fig 2C). Intracellular IL-10 expression and CD86 expression was likewise significantly upregulated in marginal zone B cells of egg- or SEA-treated mice compared to control-treated mice. SEA seemed to be more potent than egg injection in activating follicular B cells, as SEA-injection also significantly increased intracellular IL-10 and CD86 expression in this subset, although expression levels remained significantly lower compared to marginal zone B cells (Fig 2D and 2E). For analysis of B cell activation we generally restimulated cells *ex vivo* with SEA. This increased the baseline expression of CD86 in all groups compared to medium (average MFI of follicular B cells: 1013–1632±62.2; for marginal zone B cells: 2640–4118±176.9, for all groups and without significant differences between groups), but was required for detection of B cell cytokines as a result of the *in vivo* antigen exposure. Without SEA restimulation, we found a trend of increased IL-10 production by B cells which only reached significance upon additional restimulation (S1E Fig), indicating that renewed exposure to antigen is required to achieve detectable B cell activity and cytokine production. This is also supported by experiments in IL-10 GFP reporter mice, in which IL-10 (GFP) accumulates in B cells during the entire *in vivo* treatment period. Here, increased IL-10 (GFP) expression, without *ex vivo* restimulation, was only detectable in B cells of 14 weeks chronically infected mice, but not in B cells of mice that received two injections of eggs within a relatively short period of 2 weeks (S1F and S1G Fig). Altogether, these data indicate that the result of *in vivo* development of IL-10 producing B cells is in principle detectable without restimulation (S1E and S1G Fig), but the data from egg-injected IL-10 reporter mice also suggest that an *ex vivo* SEA restimulation is required to visualize its full IL-10 potential, something which will happen *in vivo* during a natural infection due to the constant production of eggs and the high levels of circulating antigens. Taken together, these data support the notion that B cells, and in particular marginal zone B cells, are responsive to *in vivo* schistosome antigen stimulation thus supporting the findings in natural schistosome infections.

### Macrophage subsets of the marginal zone bind SEA but are dispensable for Breg cell induction

**Fig 3:**
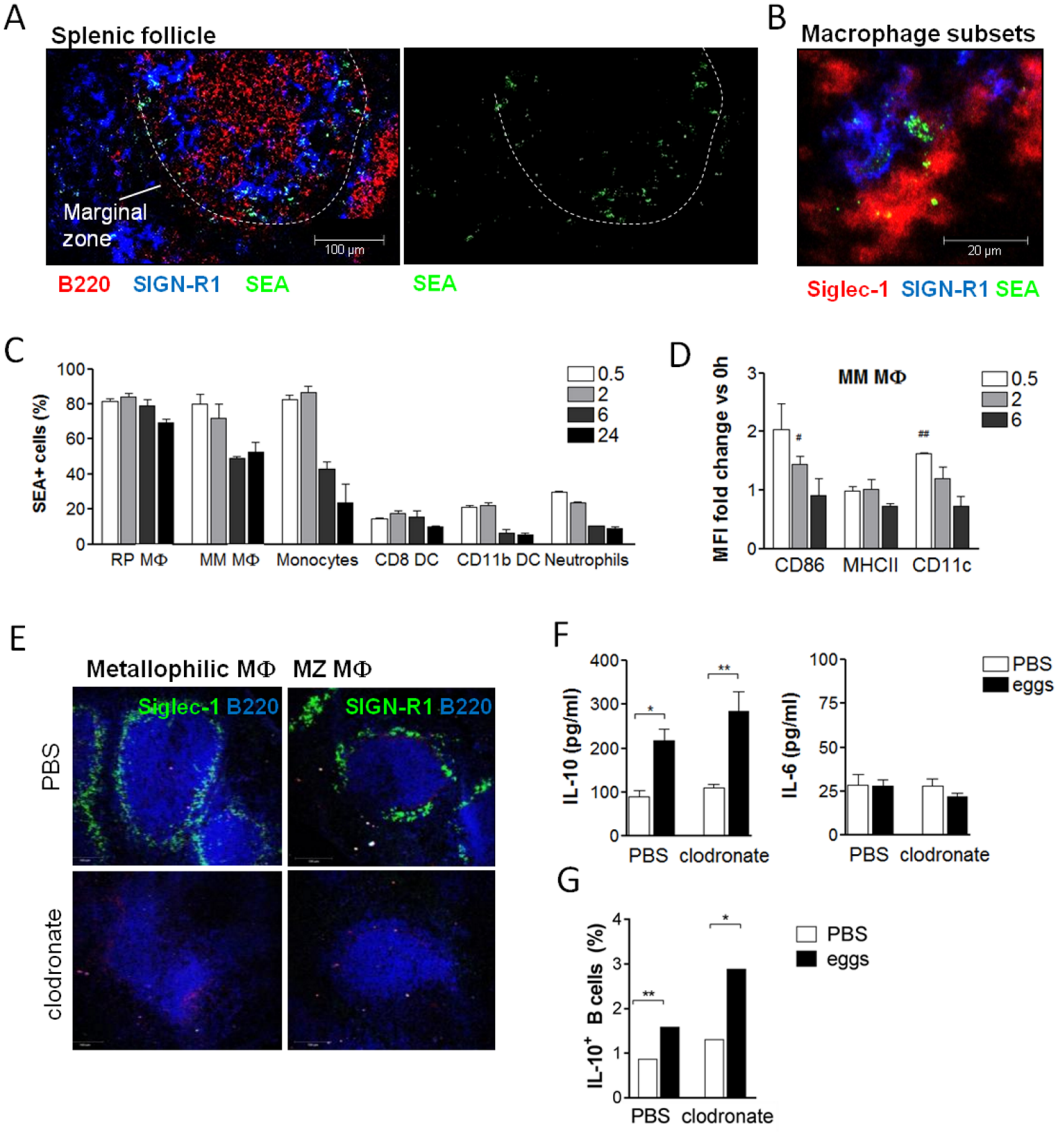
Macrophage subsets of the MZ bind SEA but are dispensable for schistosome antigen-mediated Breg cell induction. **(A, B)** Spleens were snap-frozen 30 minutes after i.v. injection of 200 μg fluorescently labeled SEA, and binding of SEA analyzed by fluorescence microscopy. Images are representative for 2 experiments with N = 5 mice and 3 follicles per section imaged. **(A)** SEA clustered around the marginal zone of the spleen (indicated by white dashed line). **(B)** SEA localized in the marginal zone to macrophages expressing Siglec-1 (marginal metallophilic macrophages) and SIGN-R1 (MZ). **(C, D)** Mice were i.v. injected with fluorescently labeled SEA and splenocytes harvested 30 minutes-24 hours later. **(C)** Frequency of SEA-positive cells in various splenocyte subsets as determined by flow cytometry (gating schemes see S4 Fig). MΦ, macrophages; RP, red pulp; MM, marginal metallophilic; DC, dendritic cells. **(D)** Mean fluorescence intensity of surface markers on MM macrophages calculated as fold increase versus the expression at time-point 0 hours. Summary of 2 experiments with N = 2. **(E-G)** Mice were i.p. injected with 200 μl clodronate-containing liposomes or PBS control liposomes. Three weeks later, mice were i.p. injected with two doses of 5000 eggs. Seven days after the last egg injection, splenic B cells were restimulated for 2 days with SEA. **(E)** Absence of splenic Siglec-1 and SIGN-R1-expressing macrophage subsets at the time-point of spleen collection was confirmed by fluorescence microscopy. B cells were stained with B220. **(F)** Cytokine concentration in culture supernatants as determined by ELISA. **(G)** Intracellular IL-10 expression of B cells after addition of Brefeldin A to the last 4 hours of the culture. One representative (N = 5) out of 2 similar experiments is shown. Significant difference by Mann-Whitney test is indicated with * p < 0.05, ** p < 0.01. Significance as determined by one-sample t-test of log-transformed data is indicated with ^#^ p < 0.05, ^##^ p < 0.01.

Molecules secreted by schistosome eggs are highly glycosylated and known to bind to C-type lectin receptors [39,40]. Since B cells show a very restricted expression of those receptors [41], we hypothesized that other C-type lectin receptor-expressing cell types in the splenic marginal zone, such as macrophages or dendritic cells, bind SEA and provide additional signals to the marginal zone B cells to support Breg cell development. Among the accessory cell types, macrophages of the splenic marginal zone were of particular interest because of their known interactions with marginal zone B cells [28,42] and schistosome antigens [43,44]. However, it was unknown whether marginal zone macrophages can capture SEA *in vivo* and are important for B cell IL-10 expression. To evaluate this, fluorescently labeled SEA was injected i.v. and 30 minutes to 24 hours later various splenic cell types were analyzed for bound SEA using fluorescence microscopy and flow cytometry. Already after 30 minutes of injection, SEA clustered along the marginal zone area of the spleen as detected by fluorescence microscopy of splenic tissue sections (Fig 3A). SEA localized predominantly within two specialized macrophage subsets of the marginal zone: SIGN-R1-expressing MZ macrophages and Siglec-1-expressing marginal metallophilic macrophages (Fig 3B). In contrast, after injection of labeled ovalbumin (OVA) as non-schistosomal control protein no fluorescence signal was detected in the spleen (S3 Fig). Flow cytometry confirmed that 83% of metallophilic macrophages were positive for SEA only 30 minutes after injection, and still 53% of cells after 24 hours (Fig 3C). In addition, F4/80-expressing red pulp macrophages and Ly6C^hi^ monocytes efficiently bound SEA (81.7 and 82.6%, respectively), while only a small fraction of dendritic cells and neutrophils did bind SEA (Fig 3C, S4A–S4C Fig for gating scheme of cell types). Metallophilic macrophages not only bound SEA abundantly, they also significantly upregulated typical surface activation markers such as CD11c and CD86, but not MHCII, at 30 minutes and 2 hours after SEA injection, respectively (Fig 3D). Because macrophages of the marginal zone were most potent in binding SEA, we next addressed their role for SEA-mediated marginal zone Breg cell induction. To this end, macrophages were depleted *in vivo* by i.p. injection of clodronate-containing liposomes [45] prior to injection of schistosome eggs. Eggs were injected 3 and 4 weeks after clodronate treatment, at time-points when only macrophages, including metallophilic and MZ subsets, were significantly reduced in spleens, but no other cell types (S4D Fig and [46]). Successful and specific depletion of splenic macrophages was also confirmed by fluorescence microscopy of tissue sections (Fig 3E) and flow cytometry (S4E Fig) at 7 days after the last egg injection, when B cell activity was analyzed. Unexpectedly, IL-10 secretion of splenic B cells from macrophage-depleted mice was equal to that from control liposome-treated mice (Fig 3F). Also the upregulation of intracellular IL-10 (Fig 3G) and CD86 expression in B cells, as well as the induction of Foxp3^+^CD25^+^ and IL-10^+^CD25^+^ CD4 T cells (S4F and S4G Fig) following SEA injection was not affected by the absence of macrophages. In conclusion, splenic macrophages are not essential for schistosome antigen-induced Breg cell development, despite the high binding of SEA by different macrophage subsets.

### MZ B cells bind and take up schistosome antigens

**Fig 4:**
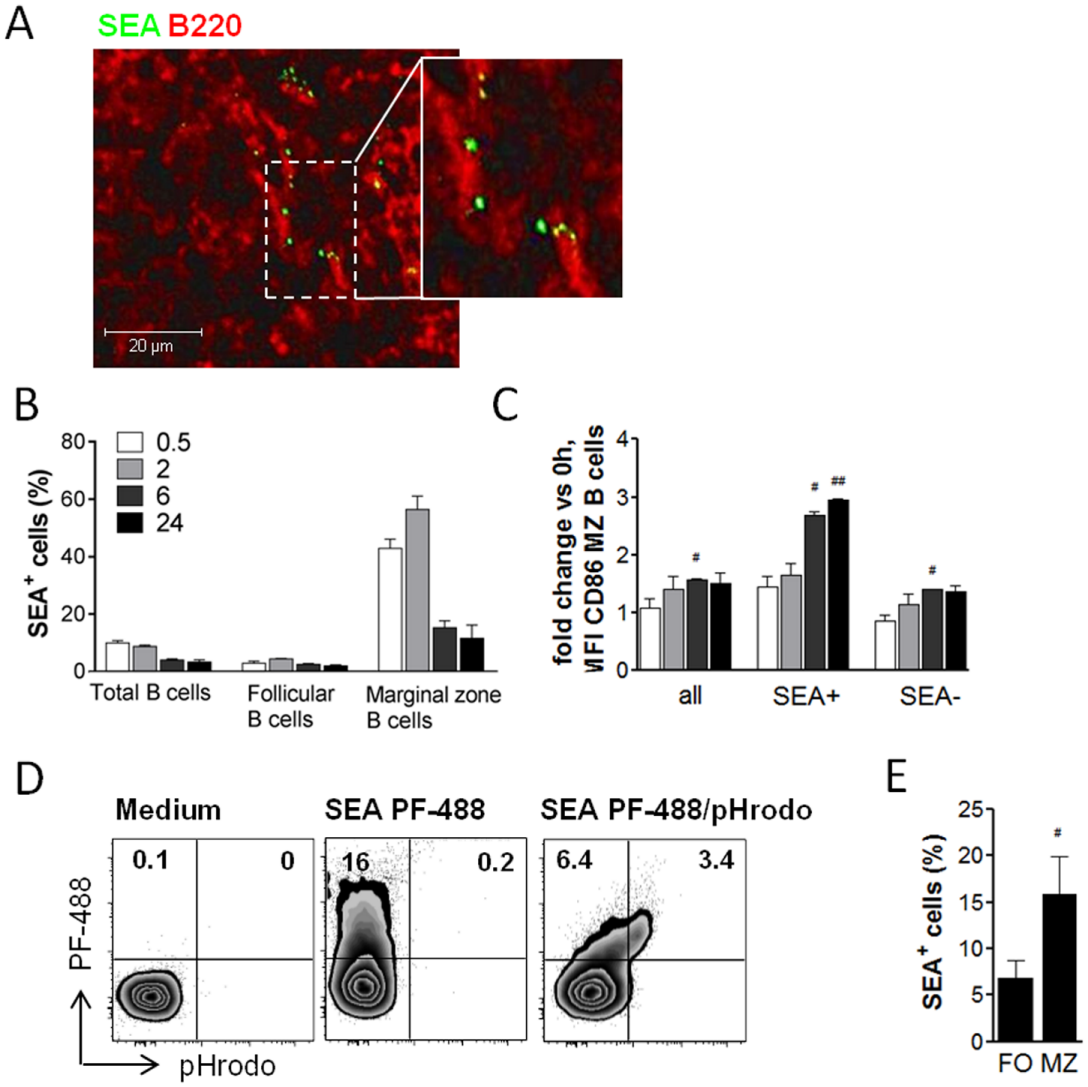
Schistosome antigens bind to B cells and are internalized into acidic compartments. **(A)** C57BL/6 mice were i.v. injected with 200 μg of fluorescently labeled SEA and spleens snap-frozen 30 minutes later. Fluorescence microscopic image shows localization of SEA to B220^+^ B cells and is representative for 2 experiments with N = 5 mice and 3 viewing fields per section imaged. **(B, C)** Splenic B cells were analyzed by flow cytometry at 30 minutes to 24 hours after i.v. injection of labeled SEA for **(B)** frequency of SEA^+^ cells within total B cells or B cell subsets as gated in Fig 2A, and for **(C)** fold increase of CD86 expression on total MZ B cells compared to MZ B cells gated for SEA^+^ and SEA^-^ populations. Summary of 2 experiments with N = 2. **(D, E)** Splenic B cells from naïve mice were cultured *in vitro* with SEA (20 μg/ml) labeled with a green dye (PF-488) alone, or co-labeled with a pH-sensitive dye (pHrodo Red). After 60 minutes, the frequency of SEA^+^ B cells was determined by flow cytometry. **(D)** Gated for total CD19^+^ B cells (1 representative out of 2 experiments shown). **(E)** Gated for follicular (FO) and marginal zone (MZ) B cell subsets (summary of 3 experiments). Significant differences indicated with ^#^ p < 0.05 and ^##^ p < 0.01 are determined by one-sample t-test of log-transformed data (C) or between B cell subsets by Wilcoxon paired test.

To test whether B cells directly bind and interact with schistosome antigens without the help of surrounding accessory cell types, fluorescently labeled SEA was injected i.v. and its co-localization with B cells analyzed by fluorescence microscopy of splenic tissue sections. Indeed, egg antigens were found to co-localize with some splenic B220^+^ B cells (Fig 4A). Flow cytometry, a more sensitive method compared to fluorescence microscopy, showed that only MZ B cells but not follicular B cells did bind SEA *in vivo*, with a maximum of 56.4% of cells being positive at 2 hours after SEA injection. SEA was still detectable on 11.5% of marginal zone B cells at 24 hours (Fig 4B). Marginal zone B cells also showed an increased CD86 surface expression following SEA injection, which was significant at 6 hours after SEA injection (Fig 4C). Interestingly, marginal zone B cells that bound SEA showed a higher CD86 expression compared to cells that were found to be negative for SEA, i.e. approximately 3-fold (SEA positive) versus only 1.5-fold (SEA negative) compared to B cells from untreated animals (Fig 4C). This suggests that marginal zone B cells not only efficiently bind egg antigens *in vivo*, but also become (more) activated as a consequence of this interaction. Binding of SEA to B cells was confirmed *in vitro* by culturing splenic B cells with fluorescently labeled SEA for 60 minutes, after which up to 16% were positive for SEA as measured by flow cytometry (Fig 4D), which is a similar percentage as found for total B cells after *in vivo* application of labeled SEA (Fig 4B). By using SEA labeled with the pH-sensitive dye pHrodo, it was shown that egg antigens were not only bound to the surface but were also internalized by B cells into acidic cellular compartments (Fig 4D). As *in vivo*, also *in vitro* the marginal zone B cell subset bound SEA more efficiently than the follicular B cell subset, with in average 15.9% versus 6.9% of cells being positive for SEA (Fig 4E). Collectively, these data show that B cells, and in particular MZ B cells, are capable of directly interacting with schistosome antigens by binding and internalization of those antigens, both *in vivo* and *in vitro*.

### Breg cells can be generated *in vitro* by direct interaction with schistosome egg antigens

**Fig 5:**
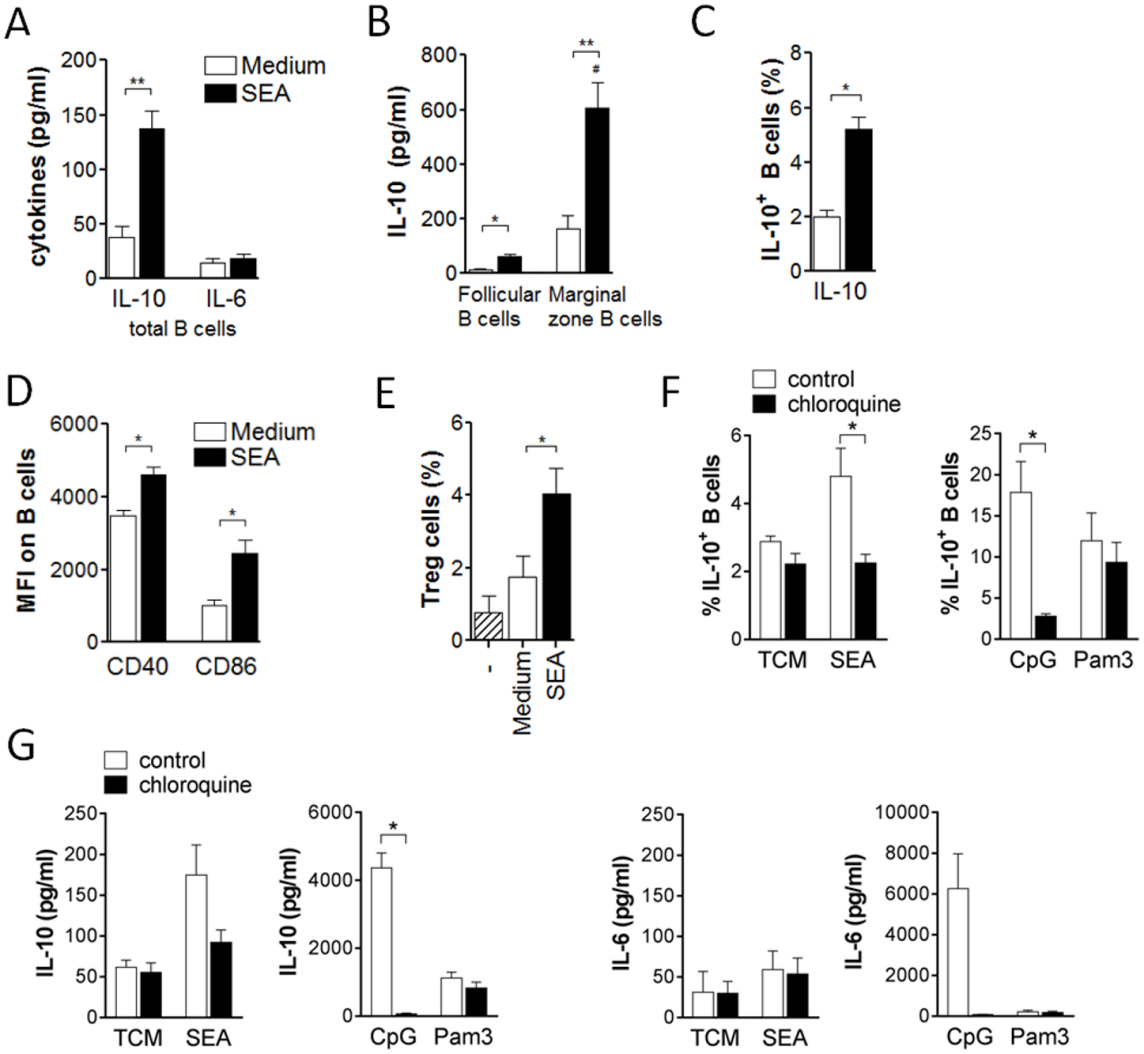
Breg cells can be generated *in vitro* by stimulation with schistosome egg antigens which involves lysosomal processing. **(A-E)** Splenic B cells from naïve mice were cultured for 3 days with 20 μg/ml SEA or medium as control. Cytokine concentration in culture supernatants of **(A)** total B cells (6 experiments) or **(B)** sorted B cell subsets (2 experiments with N = 6). **(C)** Intracellular IL-10 expression of total B cells after addition of PMA, ionomycin and Brefeldin A to the last 4 hours of the culture (4 experiments). **(D)** Mean fluorescence intensity of the activation markers CD40 and CD86 (4 experiments). **(E)** Splenic B cells were stimulated *in vitro* as above, and subsequently co-cultured with CD4^+^CD25^-^ sorted splenic T cells. After 4 days, the frequency of CD25^+^Foxp3^+^ Treg cells within the CD4 T cell population was determined by flow cytometry. Summary of 4 experiments. **(F, G)** Splenic B cells from naïve mice were cultured *in vitro* with SEA (20 μg/ml), CpG (5 μg/ml) or Pam3Cys (10 μg/ml) for 2 days. Every day, chloroquine (5 μM) was added to the culture. Intracellular IL-10 expression after addition of PMA, ionomycin and Brefeldin A during the last 4 hours of the culture **(F)**, and cytokine concentrations in culture supernatants **(G)**. Summary of 2 experiments with N = 3–4. Significant differences are indicated with * p < 0.05, ** p < 0.01 and tested by Mann-Whitney. ^#^ p < 0.05 indicates significant difference between B cell subsets by Wilcoxon paired test.

Next, we investigated whether the observed direct interaction of B cells with SEA can drive IL-10 expression and induction of regulatory B cell function *in vitro*. To this end, CD19^+^ splenic B cells from naïve mice isolated using MicroBeads were cultured for 3 days with SEA. SEA-stimulated B cells secreted significantly more IL-10, but not IL-6, compared to non-stimulated B cells (Fig 5A), showing a typical cytokine pattern characteristic for schistosome-induced Breg cells. Separate cultures of sorted MZ and follicular B cells showed once more that the MZ B cell subset reacted more potently to SEA, e.g. with a significantly higher IL-10 secretion compared to the follicular subset (Fig 5B). In addition, frequencies of B cells expressing intracellular IL-10 were likewise increased (Fig 5C). To ensure that the IL-10 phenotype is not reliant on artificially high levels of stimulation with PMA/ionomycin which is added to facilitate detection of intracellular IL-10, we repeated the assay using cells from IL-10-GFP reporter (TIGER) mice with similar results (S5 Fig). CD40 and CD86 expression were upregulated compared to cultures in medium alone (Fig 5D). Importantly, *in vitro* SEA-activated B cells were also capable of driving Treg cell development during a 4 day co-culture with CD25-depleted CD4 T cells (Fig 5E), thus providing further evidence for a bona fide regulatory function of the *in vitro* induced Breg cells. Because we had seen internalization of egg antigens into acidic compartments (Fig 4D), we wondered whether lysosomal processing is necessary for induction of IL-10 expression. Addition of chloroquine, an inhibitor of endosomal acidification [47], significantly reduced the IL-10 secretion and frequency of IL-10^+^ B cells induced by SEA and CpG (ligand for endosomal TLR9), but not by Pam3Cys (ligand for surface TLR2) (Fig 5F and 5G). This suggests that internalization and endosomal processing of SEA is required for B cell IL-10 induction. The type of receptor involved in direct activation of Breg cells by SEA remains unknown. Egg antigens are abundantly glycosylated and known to bind to C-type lectin receptors [39,40]. Le^x^-motifs, one of the most abundant glycan structures present in SEA, bind to the C-type lectin receptor SIGN-R1. However, when treating SIGN-R1-deficient mice with SEA, IL-10 expression was equally well induced compared to wild-type mice (S6A Fig), suggesting no involvement of the Le^x^-motifs. Furthermore, stimulation of various TLRs on B cells, including TLR2, TLR4, TLR7 and TLR9, has been described to induce IL-10 production [30,31]. Because SEA has been reported to contain TLR2 activity [48,49], we compared SEA-induced Breg cell responses in wild-type and TLR2-deficient B cells. We did however not observe any difference in IL-10 secretion between the two strains (S6B Fig), excluding a role of TLR2-triggering SEA components in SEA-induced B cell IL-10 production. This is further supported by the fact that TLR2-mediated B cell activation was independent of endosomal processing while SEA-mediated activation was dependent on it (Fig 5F and 5G). Collectively, these data demonstrate that Breg cells can be generated *in vitro* by culture with schistosome antigens, that endosomal processing is involved in this process, and that these Breg cells are functional in the sense that they support Treg cell development.

### CD40 ligation enhances the schistosome antigen-induced Breg cell development

**Fig 6:**
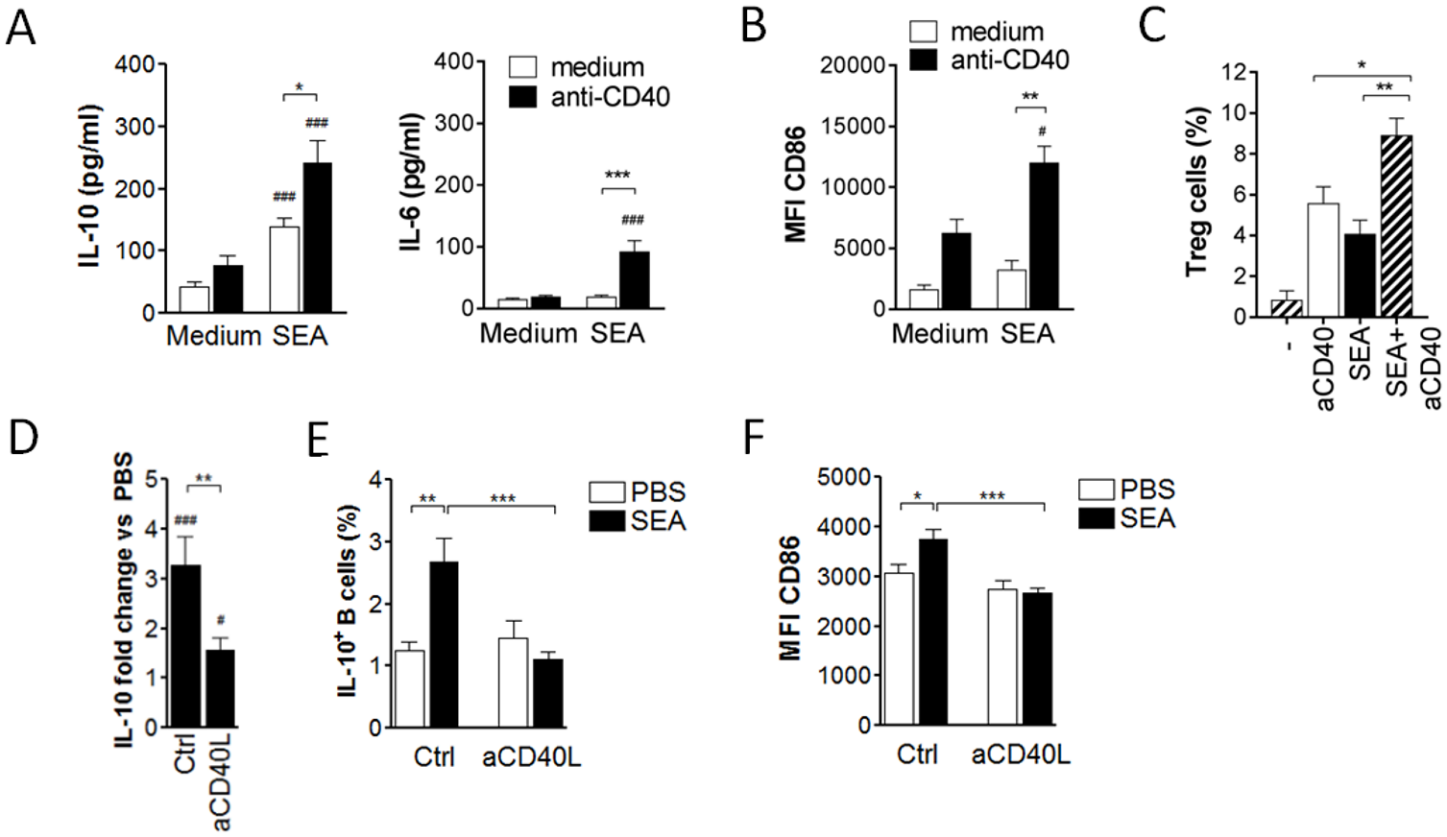
CD40 ligation enhances SEA-induced Breg cell development. **(A, B)** Splenic B cells from naïve mice were cultured with 20 μg/ml SEA or medium as control, with or without addition of anti-CD40 co-stimulatory antibody. After 3 days of culture, supernatants were analyzed for IL-10 and IL-6 by ELISA **(A)** and B cell CD86 expression by flow cytometry **(B)**. Summary of 6 experiments. **(C)** Splenic B cells were stimulated for 3 days with anti-CD40 mAb (2 μg/ml) and SEA (20 μg/ml) or anti-CD40 alone, and subsequently co-cultured with CD4^+^CD25^-^ sorted splenic T cells. After 4 days, the frequency of CD25^+^Foxp3^+^ Treg cells within the CD4 T cell population was determined by flow cytometry. Summary of 4 experiments. **(D-F)** Mice were i.p. injected with two doses of 100 μg SEA or PBS as control. CD40 ligand was blocked *in vivo* by i.p. injection of 200 μg hamster anti-mouse CD40 ligand or 200 μg hamster IgG as control (Ctrl) for 4 times, every 4 days starting at day -1 before SEA/PBS treatment. At day 14 after the first SEA/PBS injection, splenic B cells were *in vitro* restimulated with SEA for 2 days. Summary of 2 experiments (N = 8). **(D)** Fold increase of IL-10 in supernatants from B cells of SEA-injected versus PBS-injected mice. **(E)** Intracellular IL-10 expression of B cells after addition of Brefeldin A to the last 4 hours of the culture. **(F)** Mean fluorescence intensity of CD86 on B cells. Significant differences are indicated with * p < 0.05, ** p < 0.01, *** p < 0.001 as tested by Mann-Whitney test. ^#^ p < 0.05, ^##^ p < 0.01 and ^###^ p < 0.001 indicates significant difference by one-sample t-test of log-transformed data (D) or to respective medium control by Mann-Whitney test.

Previous studies highlighted a role for CD40 ligation during *in vitro* Breg cell induction [50,51]. We therefore tested whether CD40 ligation could increase the SEA-mediated effect on Breg cell development. Addition of anti-CD40 stimulatory antibody (Ab) to the 3 day SEA culture increased IL-10 secretion of splenic B cells by 1.7-fold compared to SEA alone. A similar enhancing effect was observed for IL-6 secretion (Fig 6A) and CD86 expression (Fig 6B). In contrast to anti-CD40 Ab, addition of anti-IgM Ab did not significantly enhance the SEA-mediated B cell activation (S7 Fig). To exclude stimulatory effects from LPS *in vitro*, B cells from TLR4-deficient mice were stimulated with SEA with or without addition of anti-CD40 Ab. The fold increase of IL-10 secretion compared to B cells cultured in medium was similar for wild-type and TLR4-deficient cells, thus excluding a major effect of the TLR4 ligand LPS (S6C Fig). Finally, co-culture of CD25-depleted CD4 T cells with anti-CD40-stimulated B cells increased the frequency of CD25^+^Foxp3^+^ Treg cells, which was further increased if B cells had been stimulated with SEA plus anti-CD40 Ab (Fig 6C). Thus, SEA stimulation plus CD40 ligation of B cells further enhanced the capacity to drive the development of IL-10-producing Breg cells *in vitro*.

Previous reports only addressed the role of CD40 ligation *in vitro*, but the relevance for *in vivo* Breg cell induction was not investigated. We therefore blocked CD40 ligand *in vivo* by i.p. injection of a hamster anti-mouse CD40 ligand blocking mAb (200 μg; every 4 days starting at day -1 prior to 1^st^ SEA injection) during SEA treatment of mice and analyzed the effect on Breg cell activation. In hamster IgG-injected control mice, SEA treatment increased the amount of B cell-derived IL-10 secretion by 3.3-fold compared to PBS treatment. Upon anti-CD40 ligand administration this increase was only 1.6-fold and thereby significantly lower (Fig 6D). Importantly, the SEA-mediated upregulation of intracellular IL-10 and CD86 expression by B cells was even fully abolished when CD40 ligand was blocked (Fig 6E and 6F). Taken together, CD40 ligation enhances the Breg cell-inducing effect of SEA both *in vitro* and *in vivo*.

### The secretory schistosome egg antigen IPSE/alpha-1 induces Breg cell development in mice and humans

**Fig 7:**
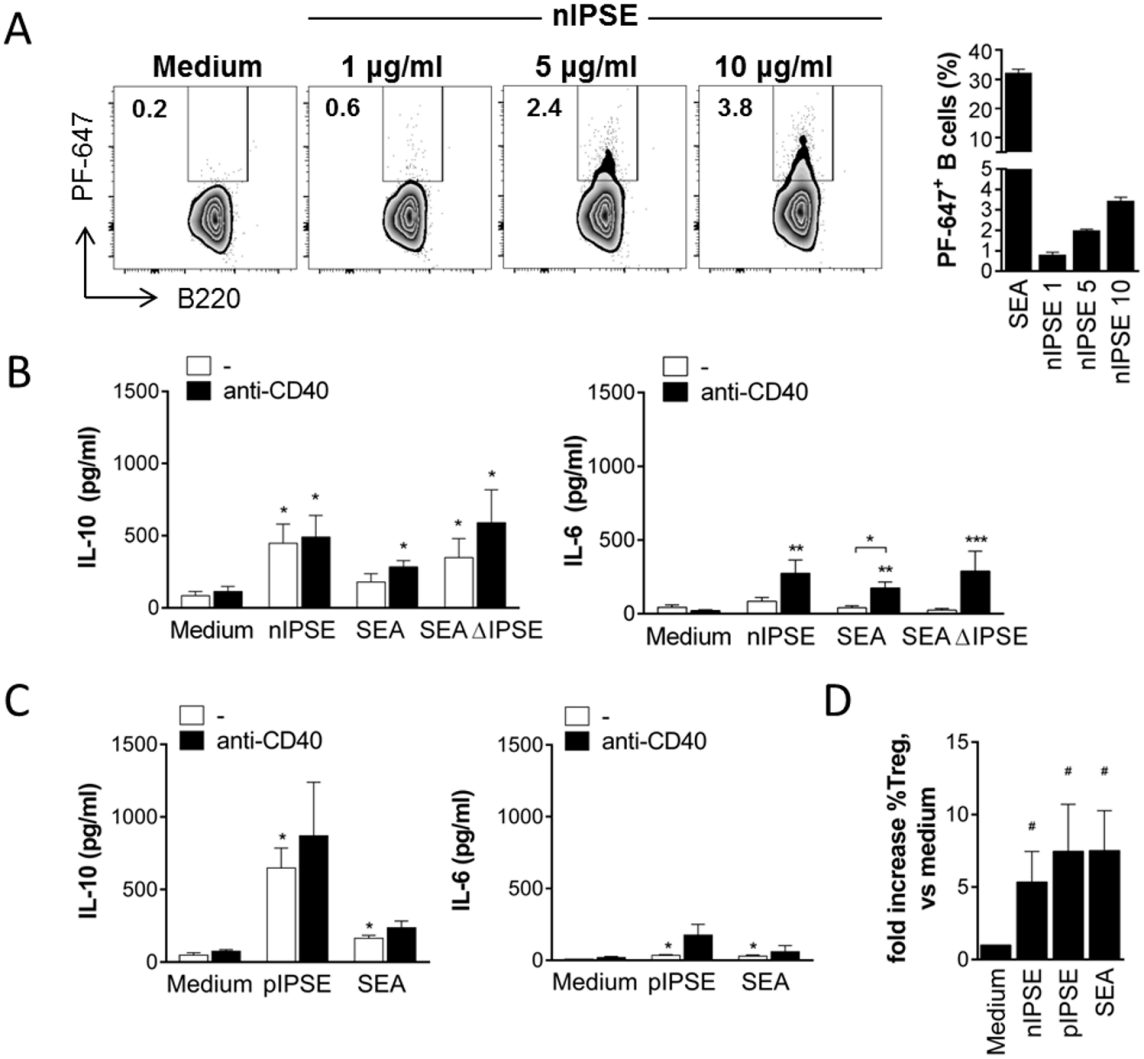
The secretory SEA component IPSE/alpha-1 induces Breg cell development. **(A)** Splenic B cells were incubated with PF-647-labeled SEA (20 μg/ml), natural IPSE/alpha-1 (nIPSE, 1–5–10 μg/ml) or left untreated and the frequency of SEA- and IPSE-positive cells measured after 60 minutes by flow cytometry. Representative FACS plots and summary of 3 experiments is shown (N = 3). **(B)** Splenic B cells from naïve mice were cultured for 3 days with 10 μg/ml nIPSE, 20 μg/ml SEA, 20 μg/ml SEAΔIPSE or medium as control, with or without addition of anti-CD40 mAb (2 μg/ml). Cytokine concentration in supernatants after 3 days as determined by ELISA. Summary of 4 experiments. **(C)** Splenic B cells from naïve mice were cultured for 3 days with 10 μg/ml recombinant, plant-derived IPSE (pIPSE), 20 μg/ml SEA or medium as control. Cytokine concentration in supernatants after 3 days as determined by ELISA. Summary of 4 experiments. **(D)** Splenic B cells were cultured for 3 days with nIPSE (10 μg/ml), SEA (20 μg/ml) or medium alone as control, and subsequently co-cultured with CD4^+^CD25^-^ sorted splenic T cells from C57BL/6 or DEREG mice. After 4 days, the frequency of CD25^+^Foxp3^+^ Treg cells within the CD4 T cell population was determined by flow cytometry. Summary of 3 experiments. Significant differences to medium control are indicated with * p < 0.05, as tested by Mann-Whitney test. ^#^ p < 0.05 indicates significant difference by one-sample t-test of log-transformed data.

**Fig 8:**
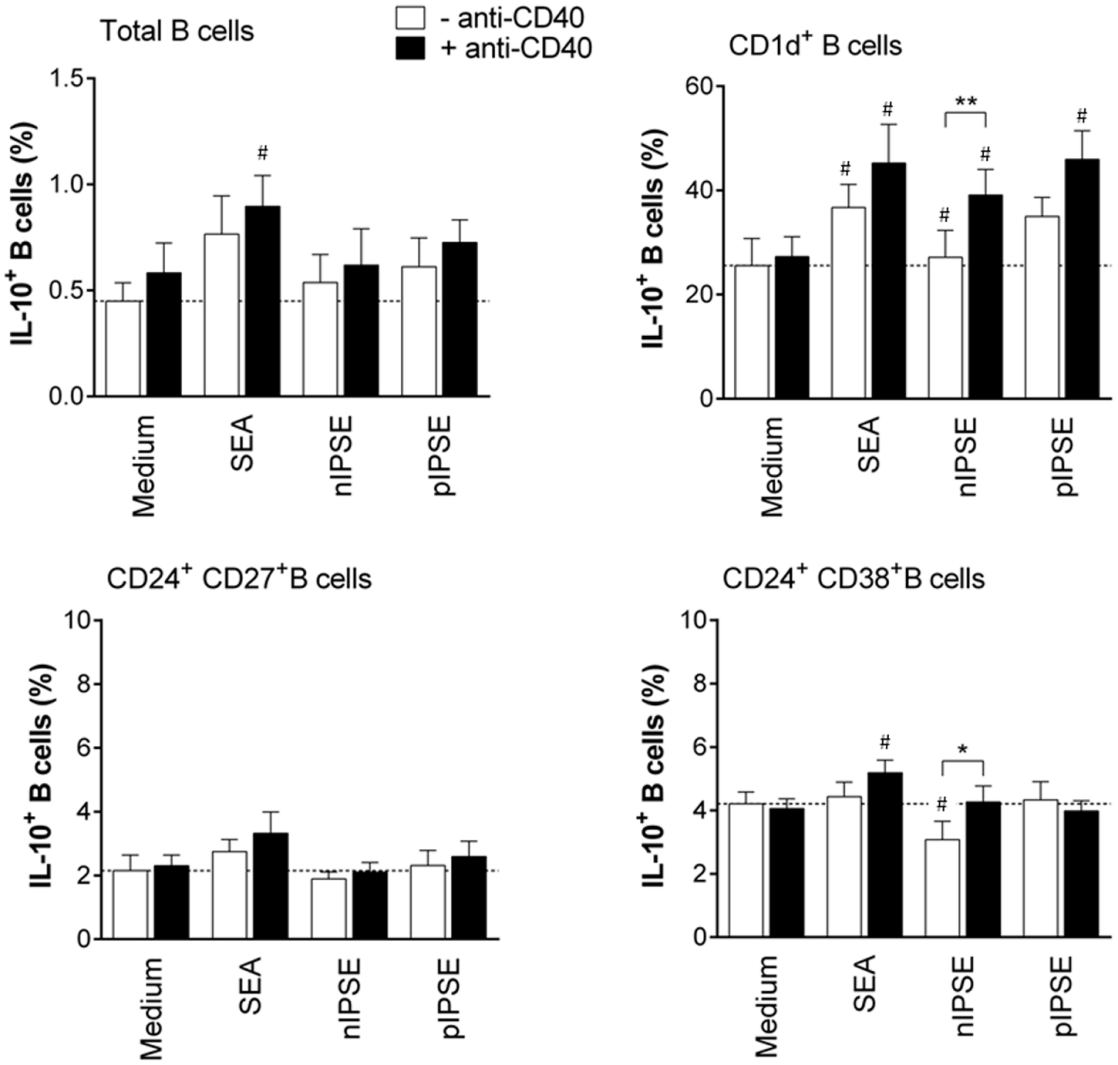
Human CD1d^+^ Breg cells increase IL-10 expression after SEA and IPSE/alpha-1 stimulation. B cells were isolated from PMBCs of healthy volunteers and stimulated for 3 days with SEA (20 μg/ml), natural IPSE (nIPSE, 10 μg/ml), plant-derived IPSE (pIPSE, 10 μg/ml), or left untreated. Different Breg cell subsets and intracellular IL-10 expression were assessed by FACS. Summary of 3 donors. Significant differences to the respective medium control are indicated with ^#^ p < 0.05, or between conditions with and without anti-CD40, with * p < 0.05, ** p < 0.01 as tested by paired t-test.

SEA is a complex mixture of several different antigens. In the next step, we therefore aimed to identify specific antigens in SEA that are relevant for Breg cell induction. We focused on three major antigens that provoke an antibody response in nearly all infected patients: omega-1, kappa-5 and IPSE/alpha-1 [52,53]. B cells were able to bind fluorescently labelled natural IPSE/alpha-1 (nIPSE), a secreted egg antigen we purified from egg extracts, in a dose-dependent manner during 60 minutes *in vitro* culture (Fig 7A). During 3 days culture however, nIPSE induced significantly elevated IL-10 but not IL-6 secretion by B cells in a concentration dependent manner (Fig 7B and S8A Fig). Importantly, recombinant IPSE/alpha-1 expressed in tobacco plants (pIPSE), which behaves as nIPSE in terms of protein dimerization and human basophil activation (S9 Fig), had similar effects to the natural molecule on B cell IL-10 and IL-6 secretion (Fig 7C). Both nIPSE and pIPSE-stimulated B cells were capable of driving Treg cell development during B cell-T cell co-culture (Fig 7D). Interestingly, SEA depleted of IPSE/alpha-1 (SEAΔIPSE) was as efficient as total SEA in inducing IL-10 secretion and CD86 expression by B cells, which suggests that also SEA antigens other than IPSE/alpha-1 can activate B cells (Fig 7B). However, as opposed to IPSE/alpha-1, other major components in SEA, such as omega-1 and kappa-5, did not increase IL-10 in any of the tested concentrations (1–20 μg/ml) (S8B Fig), thus excluding a role for these antigens in SEA-mediated Breg cell induction. Notably, omega-1 is toxic to B cells at concentrations of 5 μg/ml and above, and was therefore only tested at 1 μg/ml. For better comparability, we determined the following average relative amounts of IPSE/alpha-1, omega-1 and kappa-5 within SEA, based on the yields of several purifications of these molecules from SEA: 1.2% IPSE/alpha-1, 0.6% omega-1 and 1.8% kappa-5. Furthermore, to proof that the Breg cell-inducing effect is specific for molecules in SEA, we stimulated B cells *in vitro* with adult worm antigen (AWA) as control of an *S*. *mansoni*-derived antigen mixture not containing IPSE. AWA was unable to induce IL-10 secretion when tested in the same concentration as used for SEA (S8C Fig). For a possible future therapeutic application of antigen-activated Breg cells against e.g. allergic diseases, it is crucial to confirm the IL-10-inducing effect in human B cells. After 3 days *in vitro* stimulation with SEA and anti-CD40, we found a significant increase in the percentage of total human IL-10^+^ CD19^+^ B cells compared to cells cultured with anti-CD40 alone. Comparing different B cell subsets, which have previously been attributed with regulatory properties [54,55], we found the increase in IL-10^+^ B cells after SEA stimulation to be most pronounced among CD1d^+^ B cells rather than CD24^+^CD27^+^ and CD24^+^CD38^+^ B cells. Both nIPSE and pIPSE significantly increased the fraction of IL-10-expressing cells among CD1d^+^ B cells, whereas neither had an effect on the other two subsets investigated. As CD1d^+^ B cells only comprise a very small fraction of all B cells (CD24^+^CD27^+^ B cells >> CD24^+^CD38^+^ B cells > CD1d^+^ B cells), the effect of nIPSE and pIPSE does not translate into an increase in the percentage of IL-10^+^ cell in the total B cell pool in contrast to SEA (Fig 8). This also suggests that additional molecules in SEA may have an IL-10-inducing effect. Collectively, we demonstrated that SEA was bound to and internalized by B cells, and that this direct interaction drives the development of Breg cells. Furthermore, we identified IPSE/alpha-1 as a single molecule of SEA that induces Breg cells in mice and humans, both as a natural and a recombinant molecule.

## Discussion

In this study, we sought to identify the molecules and mechanisms involved in the induction of IL-10-producing B cells by the helminth *S*. *mansoni*. We found that soluble antigens derived from schistosome eggs, amongst which the secretory antigen IPSE/alpha-1, directly interacted with B cells. This led to the development of Breg cells characterized by IL-10 secretion and Treg cell-inducing capacity. Next to the potentially therapeutic relevance of how to generate regulatory, anti-inflammatory cells, this study also provides mechanistic insight into how schistosomes interact with the host immune system, expanding the regulatory arm of immunity and thereby prolonging its survival in the host.

While we and others have shown IL-10 expression in splenic Breg cells during natural infection with *S*. *mansoni* [8,11,12], the contribution of *S*. *mansoni*-derived egg antigens was not yet studied. SEA is highly immunostimulatory and well-known to promote Th2 as well as Treg cell responses in the host [56]. Here, we found that *S*. *mansoni* egg antigens are also able to induce IL-10-producing B cells *in vivo*, without the context of natural infection. Because SEA is a complex mixture of several different antigens, it is not unexpected that different types of immune cells and different qualities of immune responses are induced. The use of Breg cells as a therapeutic tool against inflammatory diseases is especially attractive because Breg cells in turn can induce Treg cell development [57], which would thus amplify the beneficial regulatory effect. It was therefore important to investigate whether SEA-induced IL-10-producing B cells have the capacity to trigger Treg cell development. This was indeed the case as we could show by *in vitro* co-cultures of T cells with egg antigen- or IPSE-activated B cells. Similarly, Breg cells isolated from naturally schistosome-infected mice and humans were previously shown to drive Treg cell development *in vitro* [8,12,58], thus indicating a common feature of schistosome-induced Breg cells.

Our finding that egg antigens could induce splenic Breg cell development *in vivo* raised the question whether those antigens can directly interact with B cells in the spleen. In *in vivo* binding studies using fluorescently labeled SEA and fluorescence microscopy, egg antigens were indeed found to directly bind to splenic B cells. Although various egg antigens are abundantly glycosylated, the restricted and low expression of C-type lectin receptors by B cells [41] argues rather for the involvement of non-C-type lectin PRRs expressed by B cells in the direct binding of SEA components. Indeed, preliminary experiments with B cells of SIGN-R1-deficient mice, showed an equal IL-10 expression in response to SEA compared to wild-type littermates, suggesting no involvement of the Le^x^-motifs, one of the most abundant glycan structures present in SEA and known to bind SIGN-R1. Instead, we found that within a mixture of schistosome antigens, at least the egg glycoprotein IPSE/alpha-1 was capable of driving Breg cell development *in vitro* by directly interacting with B cells, equipping them with Treg cell-inducing capacity. Two independent experimental approaches suggested that egg antigens are taken up and processed in acidic lysosomes. Previous reports on *in vitro* induction of Breg cells by helminth antigens did not use highly sort-purified B cells as in our study, but total splenocyte preparations [8,10] or merely B cell-enriched cultures [13]. Hence, it was impossible to exclude indirect, accessory cell type-mediated B cell stimulation or IL-10 production by other cell types [59,60]. Other reports addressed B cell activation by IL-10 production, but did not study the regulatory activity of schistosome antigen-exposed B cells compared to unstimulated B cells [14]. It must be emphasized that the sole demonstration of upregulated IL-10 expression is not sufficient to characterize B cells as Breg cells, as IL-10 can fulfill other roles in B cell biology independent from a regulatory function. We thus present the first report on direct induction of functional Breg cells with *in vitro* regulatory activity by helminth antigens.

With respect to the development of therapeutic applications, it would be interesting to see whether SEA-induced Breg cells are more potent than Breg cells induced by other compounds, like TLR7 or TLR9 ligands. Opposite to SEA, stimulants like R848 and CpG also induce substantial amounts of B cell proliferation and pro-inflammatory cytokines like IL-6 in addition to high levels of IL-10. It is currently unknown which side-effects would result in a therapeutic application, but it is tempting to speculate that compounds that selectively induce IL-10 are more preferable. We tried to compare IL-10-producing B cells induced *in vitro* by different agents, including SEA, for their capacity to inhibit allergic airway inflammation *in vivo*. We were however not able to confirm a suppressive capacity for any of the conditions despite the usage of a published model [31]. In the past, we have successfully applied adoptive transfers of *in vivo*, schistosome-induced Breg cells (generated during a natural infection) in allergic airway inflammation models [12]. Therefore, we assume that underlying differences between *in vivo* and *in vitro* stimulation of Breg cells may be crucial for the activity in a disease model. This may be related to issues like a differential homing or to the strength and kinetics of activation and cytokine production which determine the suppressive capacity of Breg cells on bystander immune activation in the host. Knowing that schistosome antigens can directly induce Breg cell development, we next addressed signals that regulate or enhance antigen-induced B cell IL-10 expression. In previous studies, CD40 engagement was described to induce B cell IL-10 expression [18,19,50,51,59]. This is in line with our results showing that SEA-induced IL-10 expression was significantly increased by addition of agonistic CD40 Ab. This points to the potential of a combined therapy for inflammatory diseases using helminth antigens together with anti-CD40 Ab treatment. Indeed, a report by the group of Mauri *et al*. provided evidence that experimental therapy with an agonistic Ab against CD40 can ameliorate autoimmune disease [61], as did cellular therapy with Breg cells [50], although a combined treatment was not yet tested. The groups of Fillatreau and Mauri proposed a two-step model for the acquisition of regulatory properties by B cells, with exposure to innate stimuli—such as TLR ligands—as one step and CD40 or BCR engagement as second step to establish Breg cell function [36,50]. We found a similar dependency for *in vivo* Breg induction by helminth antigen, for which CD40 ligation was crucial. Our data also show that, although Breg cell induction *in vitro* can be achieved alone without additional stimuli, engagement of CD40 further enhances this effect. Several cell types including T cells, B cells, DCs, basophils, NK cells, mast cells and macrophages express CD40 ligand (CD40L, CD154) [62] and could in principle serve as interaction partner ligating CD40 on B cells. It is however tempting to speculate that neutrophils play a role as they have been reported to express CD40L and activate MZ B cells for immunoglobulin production in a contact-dependent manner [63].

As we found MZ B cells to be the main IL-10-producing B cell subset, it was tempting to speculate that accessory cell types of the splenic marginal zone interact with schistosome antigens, and subsequently drive the development of MZ Breg cells. Macrophages of the splenic MZ were of particular interest because of their known interactions with MZ B cells during steady state [28,42] and their expression of SIGN-R1. This C-type lectin receptor was found to bind schistosome antigens *in vitro* by using SIGN-R1-overexpressing fibroblasts [43,44]. However, it was unknown whether MZ macrophages can capture SEA *in vivo* and are important for B cell IL-10 expression. As hypothesized, we found macrophages of the MZ to efficiently bind SEA upon *in vivo* administration. However, Breg cell induction was not affected upon *in vivo* depletion of macrophages, thus excluding a major role of macrophages in this process. Indirectly, also Mangan *et al*. [10] showed that macrophages were dispensable for Breg cell induction during schistosomiasis, as macrophage depletion did not affect the B cell-mediated control of anaphylaxis. The immunological role of SEA-binding MZ macrophage subsets and the identity of the binding receptor remain to be determined.

A limited number of reports is available that used specific helminth antigens to induce B cell IL-10 expression, namely the filarial antigen ES-62 [64] and the oligosaccharide lacto-N-fucopentaose III (LNFP III) that contains the Le^X^ trisaccharide antigen present on various schistosome glycoproteins [13]. Both antigens were either applied *in vivo* or used *in vitro* for stimulation of B cell-enriched cultures that still contained other cells, which means it remains unclear whether those antigens can directly bind to and interact with B cells, without indirect support from other cell types. Our study therefore identified IPSE/alpha-1 as the first helminth molecule with direct Breg cell-inducing capacity in mice and humans. Importantly, this capacity was resembled by recombinant IPSE, which is an important prerequisite for a possible therapeutic use. The use of helminth molecules for therapeutic purposes has gained renewed interest as controlled human infections with helminths showed disappointing effects in recent phase II and phase III trials (reviewed in [65]). More studies are required to define the optimal antigen, dose, time point and length of treatment as well as the suitability to treat specific inflammatory diseases [66,67]. Therefore, the identification of helminth-specific Breg-inducing antigens is warranted, even more so as the availability of active recombinant forms will ultimately allow its production under GMP conditions.

IPSE/alpha-1 was originally described as basophil IL-4-inducing principle of *Schistosoma* eggs [53] and was shown to induce a mixed Th1/Th2 type of immune response in spleen upon *in vivo* administration [68]. In our assays, IPSE/alpha-1 directly interacted with murine B cells via a still unknown receptor, which led to activation, induction of B cell IL-10 secretion and Treg cell induction *in vitro*. Although IPSE/alpha-1 is a highly glycosylated protein, we consider a role of IPSE-related glycans as unlikely because both, pIPSE and nIPSE were capable to induce B cell IL-10 expression despite differences in glycosylation (native IPSE contains Le^x^ motifs [69], while plants per definition cannot make Le^x^ motifs [70]. In addition, natural omega-1 and IPSE share a similar glycosylation (Le^x^ related) but have opposing activities both in B cells (here) and on DCs [71]. IPSE/alpha-1 has been shown to not only bind to IgE but also to IgG, both to Fc and Fab fragments [53]. We therefore hypothesize that IPSE could bind to B cells via the B cell receptor or surface-exposed IgG. Particularly important for a possible therapeutic use is our finding that both natural and plant-derived IPSE/alpha-1 induced IL-10 expression also in human CD1d^+^ Breg cells. This is even more intriguing as the CD1d^+^ B cell subset has been previously described to be increased in number and activity both in experimental infections in mice and in people living in endemic areas [12,58]. We propose a mechanism of schistosome-induced Breg cell induction in which B cells directly interact with schistosome egg antigens by binding and internalizing antigen, lysosomal processing and subsequent up-regulation of CD86 and IL-10 expression. The MZ B cell subset appeared to be particularly responsive, and CD40 engagement further enhanced Breg cell activity. Furthermore, we have successfully identified the secreted egg antigen IPSE/alpha-1 as one of the Breg-inducing antigens. These egg antigen-induced Breg cells were potent in driving Treg cell development, allowing for induction of two potent regulatory responses by the same antigen. To our knowledge, our study provides the first description of a helminth-specific molecule that interacts with and induces Breg cells, and a mechanistic insight into how schistosomes interact with their host, influence its regulatory immunity and thereby promoting their prolonged survival in the host.

## Materials and methods

### Animals

Female C57BL/6OlaHsd mice from Harlan, TLR4-deficient mice (on C57BL/6 genetic background, kindly provided by Dr. S. Akira, Osaka, Japan), TLR2-deficient mice (on C57BL/6 genetic background, kindly provided by the group of Dr. K. Willems van Dijk), SIGN-R1-deficient mice (on C57BL/6 genetic background, kindly provided by the group of Dr. W. Unger), DEREG (DEpletion of REGulatory T cells) mice (on C57BL/6 genetic background, kindly provided by Dr. T. Sparwasser) and IL-10-GFP reporter (TIGER) mice (on C57BL/6 genetic background, kindly provided by Dr. R.A. Flavell) were housed under SPF conditions in the animal facilities of the Leiden University Medical Center in Leiden, The Netherlands, and used for experiments at 8–14 weeks of age. Percutaneous infection of mice with *S*. *mansoni* was performed as described elsewhere [12], and mice sacrificed in the chronic phase (14–15 weeks) post infection.

### Preparation and purification of schistosome eggs, egg and worm antigens

Freshly isolated *S*. *mansoni* eggs from trypsinized livers of hamsters infected for 50 days were washed in RPMI medium with 300 U/ml penicillin, 300 μg/ml streptomycin (both Sigma-Aldrich, Zwijndrecht, The Netherlands) and 500 μg/ml amphotericin B (Thermo Fisher Scientific, Breda, The Netherlands) and then kept at -80°C. SEA, AWA, omega-1, kappa-5 and IPSE/alpha-1 were prepared and isolated as described previously [53,71,72,73]. The purity of the antigen preparations was checked by SDS-PAGE and silver staining, and protein concentrations determined using the BCA procedure. The antigen preparations had an endotoxin content of less than 150 ng/mg protein (SEA) or 3 ng/mg protein (purified molecules) as tested by Limulus Amoebocyte Lysate (LAL) test and TLR4-transfected HEK-reporter cell lines (kindly provided by Prof. Golenbock, University of Massachusetts Medical School, Boston, USA).

### Production and purification of recombinant IPSE from *N*. *benthamiana* plants

Recombinant IPSE was produced by transient expression in *Nicotiana benthamiana* and purified according to the methods described in [70]. In short, the complete sequence encoding the 134 AA mature *Schistosoma mansoni* IPSE (Smp_112110) was codon optimized and preceded by a signal peptide from the *Arabidopsis thaliana* chitinase gene (cSP) and a N-terminal 6x histidine-FLAG tag (H6F) was included. The full sequence was synthetically constructed at GeneArt and cloned into a pHYG expression vector. In all experiments the silencing suppressor p19 from tomato bushy stunt virus in pBIN61 was co-infiltrated to enhance expression. For gene expression the two youngest fully expanded leaves of 5–6 weeks old *N*. *benthamiana* plants were infiltrated by injecting *Agrobacterium tumefaciens* containing the IPSE expression plasmid. *N*. *benthamiana* plants were maintained in a controlled greenhouse compartment (UNIFARM, Wageningen) and infiltrated leaves were harvested at 5–6 days post infiltration. Plant-produced recombinant IPSE was obtained by applying leaf apoplast fluid containing IPSE to Ni-NTA Sepharose (IBA Life Sciences) in 50 mM phosphate buffered saline (pH 8) containing 100 mM NaCl. Bound IPSE was eluted with phosphate buffered saline (pH 8) containing 0.5M imidazole. Total soluble apoplast proteins and purified IPSE were separated under reducing/non-reducing conditions by SDS-PAGE on a 12% Bis-Tris gel (Invitrogen) and subsequently stained with Coomassie brilliant blue staining.

### Isolation of splenocytes, B cells and T cells

Single cell suspensions of murine spleens were prepared by dispersion through a 70 μm cell strainer (BD Biosciences, Breda, The Netherlands), and erythrocytes depleted by lysis. For analysis of splenic myeloid cell populations, spleens were digested for 1 hour at 37°C by incubation with collagenase D (2 mg/ml; Roche, Woerden, The Netherlands) and DNase I (2000 U/ml; Sigma-Aldrich) before dispersion. B cells were purified from spleens by using anti-CD19 MicroBeads (Miltenyi Biotec, Leiden, The Netherlands) following the manufacturer’s protocol. Purity was routinely ~95–98%. After a typical CD19 MACS sort, circa 83% of all contaminating cells were CD3^+^ T cells, 7% CD11b^+^CD11c^+^ cells, 2% CD11b^+^ CD11c^-^ cells, and 8% other cells. To determine cytokine secretion of splenic B cell subsets, CD19^+^ B cells were subsequently sorted by flow cytometry for follicular B cells (CD23^+^CD21^low^) and marginal zone B cells (CD23^-^CD21^hi^) which resulted in purities of > 98%. CD4^+^ T cells were purified from spleens by negative selection and depleted of CD25-expressing cells using anti-CD25 MicroBeads (Miltenyi Biotec).

### Isolation of human B cells from PBMCs

Peripheral blood mononuclear cells (PBMCs) were isolated from heparinized blood of healthy volunteers by Ficoll gradient centrifugation, and B cells were purified from PBMCs by using anti-CD19 MicroBeads (Miltenyi Biotec, Leiden, The Netherlands) following the manufacturer’s protocol.

### *In vitro* murine and human B cell stimulation

Mouse splenic CD19^+^ B cells (1.5x10^6^/ml) were cultured in medium (RPMI 1640 glutamax; Thermo Fisher Scientific), containing 5% heat-inactivated Fetal Bovine Serum (FBS; Greiner Bio-One, Alphen aan den Rijn, The Netherlands), 5 × 10^−5^ M 2-Mercaptoethanol (Sigma-Aldrich) and antibiotics (100 U/mL penicillin and 100 μg/mL streptomycin; Sigma-Aldrich). Human B cells (1.5 x 10^6^/ml) were cultured in medium (RPMI 1640; Thermo Fisher Scientific), containing 10% heat-inactivated Fetal Bovine Serum, pyruvate (1 mM), glutamate (2 mM) and antibiotics (100 U/mL penicillin and 100 μg/mL streptomycin; all Sigma-Aldrich). The following stimuli were added as indicated in the figure legends: SEA (20 μg/ml), SEA depleted of IPSE (SEAΔIPSE, 20 μg/ml), natural (1, 5, 10, 20 μg/ml) or plant-derived IPSE (10 μg/ml), omega-1 (1 μg/ml), kappa-5 (1, 5, 10, 20 μg/ml), AWA (5, 10, 20 μg/ml). For some conditions, co-stimulatory rat anti-mouse CD40 antibody (2 μg/ml; clone 1C10; BioLegend, Uithoorn, The Netherlands) or goat anti-mouse IgM (0.5, 1, 2 μg/ml; Jackson ImmunoResearch, Suffolk, UK) was added to the culture. After 3 days culture at 37°C, supernatants were collected for cytokine analysis by ELISA. Cells were restimulated with PMA (100 ng/ml) and ionomycin (1 μg/ml) for 4 hours in the presence of Brefeldin A (10 μg/ml; all Sigma-Aldrich) for flow cytometric analysis of intracellular IL-10. In experiments addressing involvement of lysosomal acidification, the inhibitor chloroquine (5 μM; Sigma) was added at the start of a two days culture and refreshed after 24 hours, and the TLR ligands CpG ODN 1826 (5 μg/ml; Invivogen) or Pam3Cys (10 μg/ml; Invivogen) used as control stimuli next to SEA.

### *In vivo* experiments

For *in vivo* stimulation of B cells, mice were i.p. injected with two doses of 5000 eggs or 100 μg SEA in PBS, determined as optimal doses where B cell IL-10 production plateaued in prior dose-titration experiments, and PBS or 100 μg human serum albumin (HSA) in PBS as control 7 days apart. At day 14 after the first injection, splenic B cells were harvested and cultured in medium at 1.5 x 10^6^ cells/ml or restimulated for 2 days with SEA (20 μg/ml) to allow detection of cytokines, as established for *in vivo* schistosome-exposed B cells before [12]. Supernatants were collected to determine cytokine concentration by ELISA. Cells were cultured for additional 4 hours with Brefeldin A (10 μg/ml; Sigma-Aldrich) to detect intracellular IL-10 by flow cytometry. In some experiments, mice were treated i.p. with 200 μg of hamster anti-mouse CD40 ligand blocking antibody (clone: MR1) or 200 μg hamster IgG as control (Jackson ImmunoResearch) 4 times, every 4 days starting one day before SEA or PBS treatment. For *in vivo* depletion of macrophages, mice were i.p. injected with 200 μl clodronate-containing liposomes and control mice with 200 μl of PBS liposomes (ClodronateLiposomes.com, Amsterdam, The Netherlands) [45] three weeks prior to egg antigen treatment. Successful and specific depletion of splenic macrophage subsets was confirmed by fluorescence microscopy and flow cytometry.

### Co-culture with CD4^+^CD25^-^ T cells

*In vitro* or *in vivo* SEA-stimulated CD19^+^ B cells were co-cultured with MACS-sorted CD4^+^CD25^-^ T cells at 1:1 ratio (each 1 x 10^6^/ml) to test for *in vitro* Treg cell induction. After 4 days, Treg cell frequencies were determined by flow cytometry by gating for Foxp3^+^CD25^+^ cells in the CD3^+^CD4^+^ T cell population, and culture supernatants collected for subsequent ELISA.

### Antigen binding assays

SEA, IPSE/alpha-1 and ovalbumin (OVA) were fluorescently labeled with PF-488 or PF-647 using the PromoFluor labeling kits (PromoCell, Heidelberg, Germany) according to the manufacturer’s protocol. For some experiments, SEA was co-labeled with the pH-sensitive pHrodo Red dye (Thermo Fisher Scientific). After protein labeling, non-reacted dye was removed using Zeba desalt spin columns (Thermo Fisher Scientific). For analysis of binding *in vitro*, CD19^+^ splenic B cells were cultured for 60 minutes at 37°C with 20 μg/ml fluorescently labeled SEA or 1–10 μg/ml of IPSE antigen, then washed in ice-cold PBS before analysis by flow cytometry. For analysis of *in vivo* binding, mice were i.v. injected with 200 μg of fluorescently labeled SEA or OVA as non-schistosomal control protein and spleens snap-frozen 30 minutes to 24 hours later. Binding of SEA to B cells was analyzed by confocal fluorescence microscopy of tissue sections and by flow cytometry.

### Induction of IL-4 release from human basophils

Basophils were purified from 250 ml of peripheral blood of healthy human donors to a mean purity of 99% by a three-step protocol consisting of a density gradient centrifugation via Ficoll/Percoll (100/6, density 1.080 g/l), followed by enrichment of the basophils via counter flow elutriation and final purification by magnetic cell sorting using the basophil isolation kit II for negative selection of basophils (Miltenyi-Biotech). Purified basophils were cultured in Iscove's Modified Dulbecco's Media (IMDM; PAA) containing 2 mM glutamine (PAA), 5 μg/ml insulin (Gibco), 50 μg/ml apo-transferrin (Sigma-Aldrich), 100 μg/ml Pen/Strep (PAA), 10% heat-inactivated Fetal Calf Serum (FCS-Gold; PAA) and 2.5 ng/ml IL-3 (kind gift of Kirin Brewery, Japan). Basophils were pre-incubated for regeneration for 30 min at 37°C, 6% CO_2_, and then stimulated at a concentration of 0.025 x 10^6^ basophils /ml in 96well flat-bottom culture plates in 100 μl at 37°C, 6% CO_2_. Concentration of stimuli was as indicated. Culture supernatants were collected after 18h and stored at -20°C.

### Flow cytometry

Flow cytometric analysis of murine B cells was performed by staining with fluorochrome-labeled antibodies against CD19, CD21 (both BD Biosciences), CD23, CD40, CD86 or IL-10 (all eBioscience) after fixation with 1.9% paraformaldehyde and permeabilization with 0.5% saponin (Sigma-Aldrich). Human B cells were stained for CD19, CD38 (both BD Biosciences), CD24, CD27 (both eBioscience), CD1d, IL-10, TNF (all Biolegend), and CD39 (Sony Biotechnology, San Jose, USA). Splenic myeloid cell subsets were discriminated using fluorochrome-labeled antibodies against CD11b, CD11c (both eBioscience), CD8, Ly6C (both Biolegend), F4/80 (AbD Serotec, Puchheim, Germany), Gr-1 (BD Biosciences), and Siglec-1 (Dr. J. den Haan, VUMC, Amsterdam, The Netherlands). Treg cells were fixed and permeabilized with the eBioscience Foxp3 fixation/permeabilization kit and stained using fluorochrome-labeled antibodies against CD3, CD4, Foxp3 (all eBioscience) and CD25 (BD Biosciences). All cells were stained with Aqua dye (Thermo Fisher Scientific) prior to fixation to discriminate dead cells. For all flow cytometric stainings, FcγR-binding inhibitor (2.4G2) was added and FMOs were used for gate setting. Flow cytometry was performed using a FACSCanto or Fortessa (BD Biosciences).

### ELISA

The concentration of murine IL-6 and IL-10 as well as human IL-4 present in culture supernatants was quantified by commercial ELISA kits according to the manufacturer’s instructions (BD Biosciences or Eli-Pair, Diaclone).

### Confocal microscopy

Spleens were snap-frozen in O.C.T. medium (Tissue-Tek; Sakura, Alphen aan den Rijn, The Netherlands). Cryosections (10 μm) were fixed in ice cold acetone for 10 minutes, air-dried, and blocked in 1% BSA plus 20% FBS in PBS before staining with Abs at room temperature. Cryosections were incubated with rat anti-mouse Siglec-1 (clone SER-4; provided by Dr. J. den Haan, VUMC, Amsterdam, The Netherlands) followed by Alexa555-conjugated goat anti-rat IgG (Invitrogen), anti-SIGN-R1 Alexa647 (clone 22D1; Dr. J. den Haan) and anti-B220 eFluor450 (eBioscience). Images were acquired using a Zeiss LSM 710 confocal laser scanning microscope with Zen software (Carl Zeiss Microimaging, Jena, Germany).

### Statistical analysis

All data are presented as mean ± standard error of the mean (SEM). Statistical analysis was performed with GraphPad Prism version 6.00 for Windows (GraphPad Software, La Jolla, CA, USA) using nonparametric Mann-Whitney U test to compare different groups and Wilcoxon paired test to compare B cell subsets. One-sample t-test of log-transformed data was applied to calculate significant changes for data which are expressed as fold increase. All p-values < 0.05 were considered significant.

### Ethics statement

All animal studies were performed in accordance with the Animal Experiments Ethical Committee of the Leiden University Medical Center (DEC-12204). The Dutch Experiments on Animals Act is established under European Guidelines (EU directive no. 86/609/EEC regarding the Protection of Animals used for Experimental and Other Scientific Purposes). For the isolation of B cells from PBMCs, human subjects were recruited within the framework of the study P09.170, which was approved by the Medical Ethical Committee of the Leiden University Medical Center. For the isolation of basophils, donors were recruited under approval by the Ethics Committee of the University of Luebeck (AZ-12-202A). Studies were performed according to the declaration of Helsinki and all participants were adults and have given written informed consent.

## Supporting information

S1 Fig Additional data on the specificity and persistence of egg antigen-induced B cell activity. **(A-B)** A non-schistosomal control protein does not activate B cells. Wild-type mice were i.p. injected with two doses of 100 μg SEA in PBS, 100 μg human serum albumin (HSA) in PBS or PBS alone. At day 14, CD19^+^ sorted splenic B cells were restimulated with SEA (20 μg/ml) for 2 days. **(A)** IL-10 concentration in culture supernatant as determined by ELISA. **(B)** Mean fluorescence intensity of CD86 expression as determined by FACS. One out of 2 similar experiments is shown. **(C)** Intracellular IL-10 expression of splenic B cells isolated from C57BL/6 mice infected chronically (14 weeks) with *S*. *mansoni*, compared to naïve control mice. **(D)** IL-10-producing B cells can be detected at least 28d after egg injection. Wild-type mice were injected twice with 5000 *S*. *mansoni* liver eggs (d-7, d0) by i.p. injection. Splenic B cells were isolated on d7, d14 and d28 after the last egg injection and re-stimulated *in vitro* with SEA (20 μg/ml) for 2 days. Supernatants were harvested and the concentration of IL-10 determined by ELISA. One experiment with n = 5 mice per group. * p < 0.05 as determined by students t-test. **(E)** Wild-type mice were treated twice with SEA (100 μg) or PBS by i.p. injection. On day 14, splenic B cells were isolated and re-stimulated for 2 days with SEA or left untreated (medium). IL-10 concentration in culture supernatants as assessed by ELISA. Summary of 5 experiments. **(F, G)** IL-10 reporter (TIGER) mice were treated twice with 5000 *S*. *mansoni* eggs **(F)** or infected with *S*. *mansoni* until the chronic phase of infection (14 weeks) **(G)**. The percentage of IL-10-GFP+ total B cells or follicular (FO) and marginal zone (MZ) B cells within the spleen as assessed by FACS. Summary of three experiments with N = 15 mice per group (F) or one experiment with N = 2–4 mice per group (G). Significant differences are indicated with * p < 0.05 and ** p < 0.01 as tested by Mann-Whitney test.
(TIF)

S2 Fig B cell IL-10 induction by egg antigens is independent of TLR4. C57BL/6 wild-type and TLR4-deficient mice were i.p. injected with two doses of 100 μg SEA in PBS, or PBS as control. At day 14, CD19^+^ sorted splenic B cells were restimulated with SEA (20 μg/ml) for 2 days. Secreted IL-10, intracellular IL-10 and CD86 expression of B cells are shown. Significant differences are indicated with * p < 0.05, ** p < 0.01, *** p < 0.001 as tested by Mann-Whitney test.
(TIF)

S3 Fig Macrophage subsets of the MZ do not bind the control protein ovalbumin. Spleens were snap-frozen 30 minutes after i.v. injection of 200 μg fluorescently labeled SEA or ovalbumin (OVA), and binding analyzed by fluorescence microscopy. SEA but not OVA localized in the marginal zone to macrophages expressing Siglec-1 (marginal metallophilic macrophages) and SIGN-R1 (MZ macrophages). Images are representative of N = 5 mice and 3 follicles per section imaged.
(TIF)

S4 Fig Depletion control for splenic cell subsets and Treg cell activity after clodronate treatment. **(A-C)** Gating scheme and representative FACS plots of splenocyte subsets which were subsequently analyzed for *in vivo*-captured fluorescently labeled egg antigens (shown in Fig 3). Splenocytes were pre-gated for living singlets. **(A)** Red pulp macrophages were gated as F4/80^+^CD11b^int^, marginal metallophilic (MM) macrophages are Siglec-1-positive and highly autofluorescent. **(B)** Dendritic cells were gated CD11c^hi^CD11b^int/+^ and further divided into CD8^+^CD11b^int^ and CD8^-^CD11b^+^ subsets. **(C)** Monocytes were gated as CD11b^+^Ly6C^hi^Gr-1^int^, neutrophils as CD11b^+^Ly6C^int^Gr-1^hi^. **(D)** Frequency of splenic cell types before (0 days) and at 1–28 days after i.p. injection of clodronate-containing liposomes. Only macrophage frequencies were significantly reduced at day 21 and 28. **(E)** Frequency of splenic cell types at the time-point of B cell analysis in egg-treated mice (day 7 after the second egg injection, i.e. day 35 after clodronate treatment). **(F, G)** Macrophage depletion prior to SEA treatment does not change the frequency of Treg cells. Mice were treated with chlodronate and injected with SEA i.p. The percentage of Foxp3^+^ CD25^+^ Treg cells in splenocytes **(F)** and IL-10^+^ CD25^+^ T cells **(G)** as assessed by FACS. Summary of 2 experiments with N = 4–6 mice per group (D), N = 6–12 (E), or data from one experiment with N = 5 mice per group (F, G). Significant differences to the respective control (D, day 0; E-G, PBS or eggs) are indicated with ^#^ p < 0.05, ^##^ p < 0.01, ^###^ p < 0.001 as obtained by Mann-Whitney test.
(TIF)

S5 Fig The IL-10 phenotype is not reliant on artificially high stimulation with PMA/ionomycin. Splenic B cells from IL-10-GFP (TIGER) mice were cultured for 2 days with 20 μg/ml SEA or medium as control. Intracellular IL-10 expression of total B cells as assessed by GFP signal in the presence or absence of PMA and ionomycin during the last 4 hours of the culture. Data are from 1 experiment with N = 5 per group.
(TIF)

S6 Fig B cell IL-10 induction by SEA is independent of SIGN-R1, TLR2 and TLR4. **(A)** C57BL/6 wild-type or SIGN-R1-deficient mice were treated with 2 doses of SEA (each 100 μg) for 2 weeks or PBS as control. Secreted IL-10 was detected by ELISA after 2 days restimulation of splenic CD19^+^ B cells with SEA (20 μg/ml). Summary of N = 2–6 mice per group. **(B, C)** Splenic B cells from naïve wild-type, TLR2-deficient **(B)** and TLR2-deficient **(C)** mice were cultured for 3 days with 20 μg/ml SEA or medium as control, with or without addition of anti-CD40 (0.5 μg/ml). IL-10 concentration in culture supernatants is expressed as fold increase versus the medium or anti-CD40 control in a summary of 3 experiments. Significance was tested by one-sample t-test of log-transformed data and is indicated by ^#^ p < 0.05, ^##^ p < 0.01.
(TIF)

S7 Fig Anti-IgM Ab does not further increase SEA-induced IL-10 expression of B cells. Splenic B cells from naïve mice were cultured with 20 μg/ml SEA or medium as control, with or without addition of anti-IgM Ab in different concentrations. After 3 days of culture, supernatants were analyzed for IL-10 and IL-6 by ELISA. Summary of 3 experiments. Significant differences are indicated with ** p < 0.01 and *** p < 0.001 as tested by Mann-Whitney test.
(TIF)

S8 Fig IPSE/alpha-1 activates Breg cells dose-dependently while the major egg antigens omega-1 and kappa-5 as well as worm antigen are ineffective. Splenic B cells from naïve mice were cultured for 3 days with different concentrations (indicated by numbers in the x-axis label) of natural (n) IPSE/alpha-1, omega-1 or kappa-5, or medium as negative and 20 μg/ml SEA as positive control. **(A)** IL-10 and IL-6 concentration in culture supernatants after nIPSE stimulation as measured by ELISA. Average of duplicates from one experiment shown. **(B)** IL-10 concentration in supernatants of kappa-5 or omega-1 stimulated B cells. Average of duplicates from one experiment shown. **(C)** Splenic B cells were stimulated *in vitro* with SEA (20 μg/ml), anti-CD40 (2 μg/ml) or adult worm antigen AWA (5–10–20 μg/ml) for 3 days. IL-10 in culture supernatant as determined by ELISA. Average of duplicate values from one experiment shown.
(TIF)

S9 Fig Plant-based production of IPSE. **(A)** SDS-PAGE and Coomassie blue staining of apoplast fluids (AF) from empty vector (EV), N-terminally tagged H6F-IPSE infiltrated *N*. *benthamiana* plants and subsequent small-scale purification of IPSE using Ni-NTA resin and the Äkta Prime purification system. Purified IPSE was analysed under reducing and non-reducing conditions (± DTT). **(B)** IL-4 release from isolated human basophils during 18h stimulation with pIPSE and nIPSE (0.16–2500 ng/ml), as determined by ELISA. Data are presented as percentage of maximum IL-4 release. Summary of N = 4–6 donors.
(TIF)
