# Supplementary figures and images for: Schistosome egg antigens, including the glycoprotein IPSE/alpha-1, trigger the development of regulatory B cells
Source: PLoS Pathog. 2017 Jul 28;13(7):e1006539. doi: 10.1371/journal.ppat.1006539 (PMC5550006; doi:10.1371/journal.ppat.1006539)

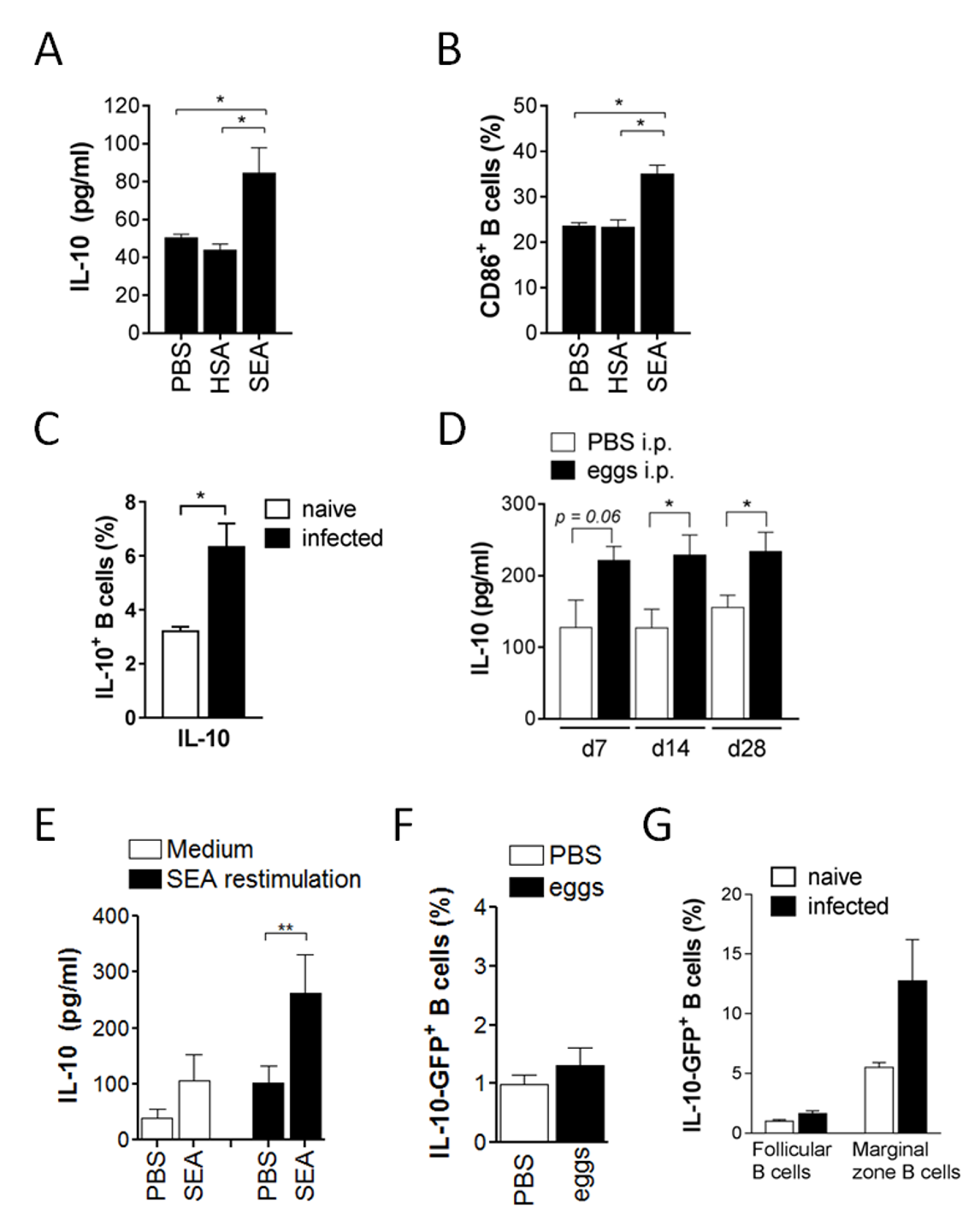

Supplement: S1 Fig — (A-B) A non-schistosomal control protein does not activate B cells. Wild-type mice were i.p. injected with two doses of 100 μg SEA in PBS, 100 μg human serum albumin (HSA) in PBS or PBS alone. At day 14, CD19+ sorted splenic B cells were restimulated with SEA (20 μg/ml) for 2 days. (A) IL-10 concentration in culture supernatant as determined by ELISA. (B) Mean fluorescence intensity of CD86 expression as determined by FACS. One out of 2 similar experiments is shown. (C) Intracellular IL-10 expression of splenic B cells isolated from C57BL/6 mice infected chronically (14 weeks) with S. mansoni, compared to naïve control mice. (D) IL-10-producing B cells can be detected at least 28d after egg injection. Wild-type mice were injected twice with 5000 S. mansoni liver eggs (d-7, d0) by i.p. injection. Splenic B cells were isolated on d7, d14 and d28 after the last egg injection and re-stimulated in vitro with SEA (20 μg/ml) for 2 days. Supernatants were harvested and the concentration of IL-10 determined by ELISA. One experiment with n = 5 mice per group. * p < 0.05 as determined by students t-test. (E) Wild-type mice were treated twice with SEA (100 μg) or PBS by i.p. injection. On day 14, splenic B cells were isolated and re-stimulated for 2 days with SEA or left untreated (medium). IL-10 concentration in culture supernatants as assessed by ELISA. Summary of 5 experiments. (F, G) IL-10 reporter (TIGER) mice were treated twice with 5000 S. mansoni eggs (F) or infected with S. mansoni until the chronic phase of infection (14 weeks) (G). The percentage of IL-10-GFP+ total B cells or follicular (FO) and marginal zone (MZ) B cells within the spleen as assessed by FACS. Summary of three experiments with N = 15 mice per group (F) or one experiment with N = 2–4 mice per group (G). Significant differences are indicated with * p < 0.05 and ** p < 0.01 as tested by Mann-Whitney test. (TIF) [file ppat.1006539.s001.tif]

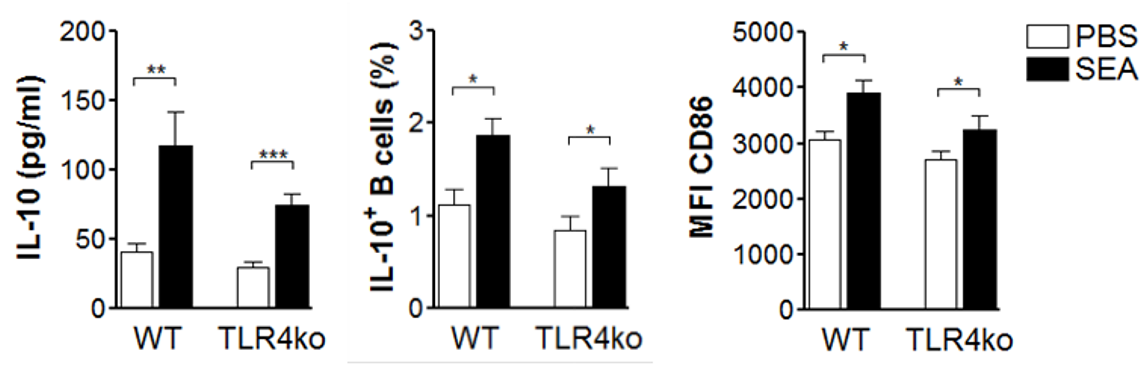

Supplement: S2 Fig — C57BL/6 wild-type and TLR4-deficient mice were i.p. injected with two doses of 100 μg SEA in PBS, or PBS as control. At day 14, CD19+ sorted splenic B cells were restimulated with SEA (20 μg/ml) for 2 days. Secreted IL-10, intracellular IL-10 and CD86 expression of B cells are shown. Significant differences are indicated with * p < 0.05, ** p < 0.01, *** p < 0.001 as tested by Mann-Whitney test. (TIF) [file ppat.1006539.s002.tif]

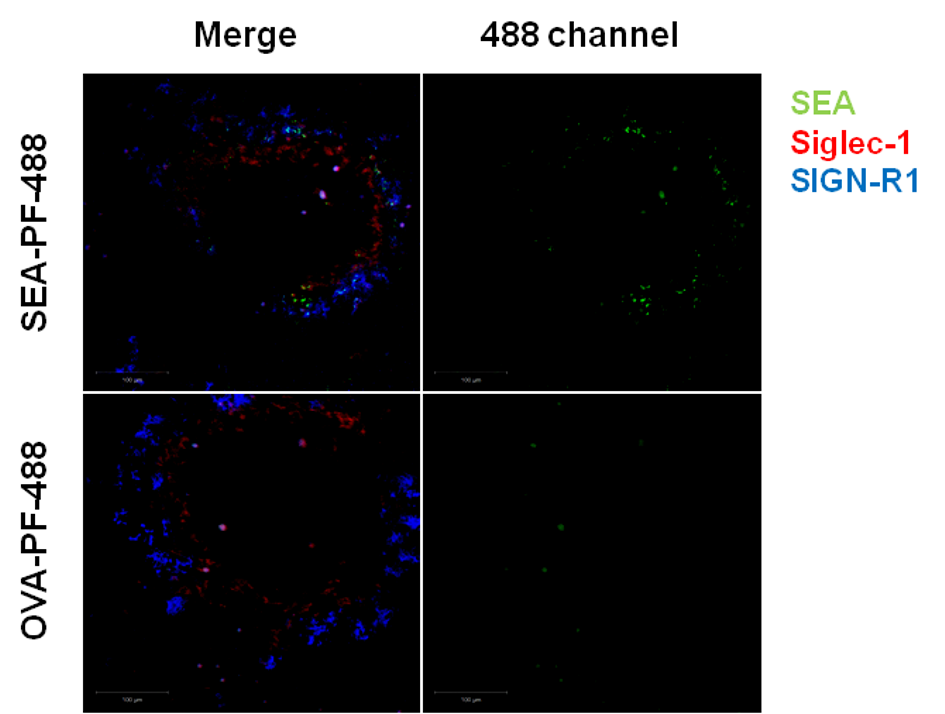

Supplement: S3 Fig — Spleens were snap-frozen 30 minutes after i.v. injection of 200 μg fluorescently labeled SEA or ovalbumin (OVA), and binding analyzed by fluorescence microscopy. SEA but not OVA localized in the marginal zone to macrophages expressing Siglec-1 (marginal metallophilic macrophages) and SIGN-R1 (MZ macrophages). Images are representative of N = 5 mice and 3 follicles per section imaged. (TIF) [file ppat.1006539.s003.tif]

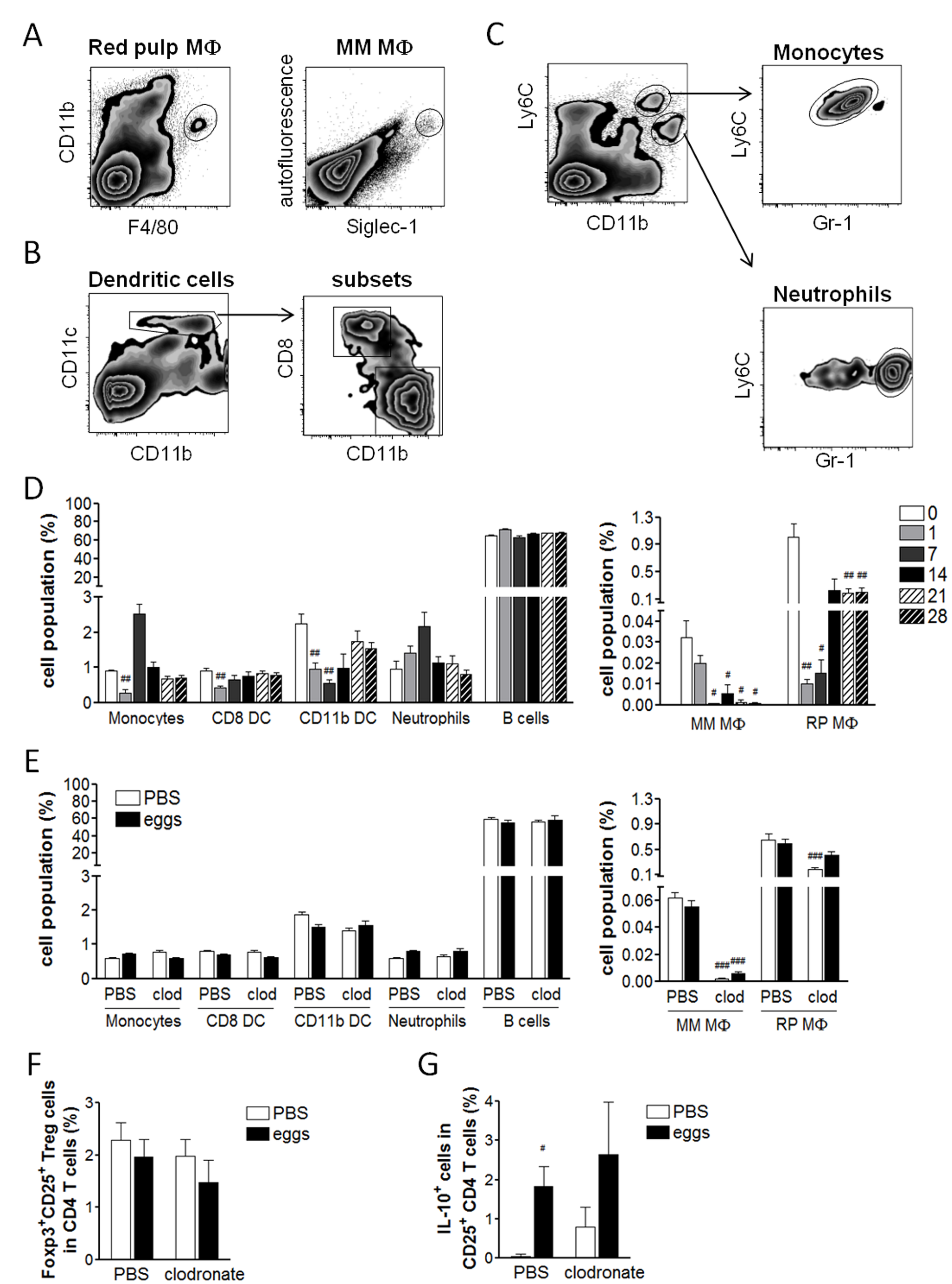

Supplement: S4 Fig — (A-C) Gating scheme and representative FACS plots of splenocyte subsets which were subsequently analyzed for in vivo-captured fluorescently labeled egg antigens (shown in Fig 3). Splenocytes were pre-gated for living singlets. (A) Red pulp macrophages were gated as F4/80+CD11bint, marginal metallophilic (MM) macrophages are Siglec-1-positive and highly autofluorescent. (B) Dendritic cells were gated CD11chiCD11bint/+ and further divided into CD8+CD11bint and CD8-CD11b+ subsets. (C) Monocytes were gated as CD11b+Ly6ChiGr-1int, neutrophils as CD11b+Ly6CintGr-1hi. (D) Frequency of splenic cell types before (0 days) and at 1–28 days after i.p. injection of clodronate-containing liposomes. Only macrophage frequencies were significantly reduced at day 21 and 28. (E) Frequency of splenic cell types at the time-point of B cell analysis in egg-treated mice (day 7 after the second egg injection, i.e. day 35 after clodronate treatment). (F, G) Macrophage depletion prior to SEA treatment does not change the frequency of Treg cells. Mice were treated with chlodronate and injected with SEA i.p. The percentage of Foxp3+ CD25+ Treg cells in splenocytes (F) and IL-10+ CD25+ T cells (G) as assessed by FACS. Summary of 2 experiments with N = 4–6 mice per group (D), N = 6–12 (E), or data from one experiment with N = 5 mice per group (F, G). Significant differences to the respective control (D, day 0; E-G, PBS or eggs) are indicated with # p < 0.05, ## p < 0.01, ### p < 0.001 as obtained by Mann-Whitney test. (TIF) [file ppat.1006539.s004.tif]

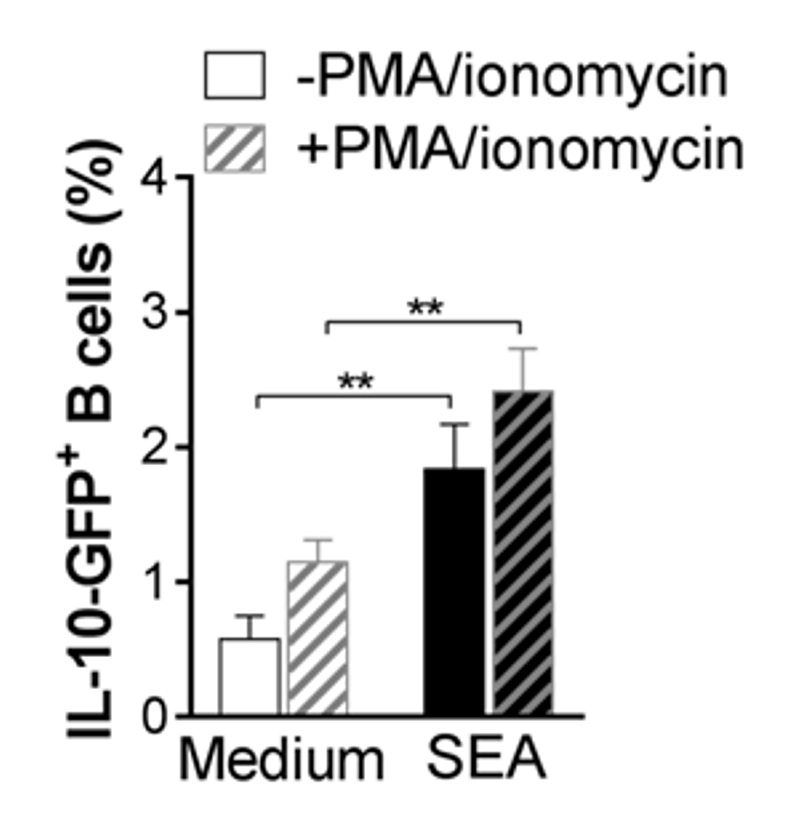

Supplement: S5 Fig — Splenic B cells from IL-10-GFP (TIGER) mice were cultured for 2 days with 20 μg/ml SEA or medium as control. Intracellular IL-10 expression of total B cells as assessed by GFP signal in the presence or absence of PMA and ionomycin during the last 4 hours of the culture. Data are from 1 experiment with N = 5 per group. (TIF) [file ppat.1006539.s005.tif]

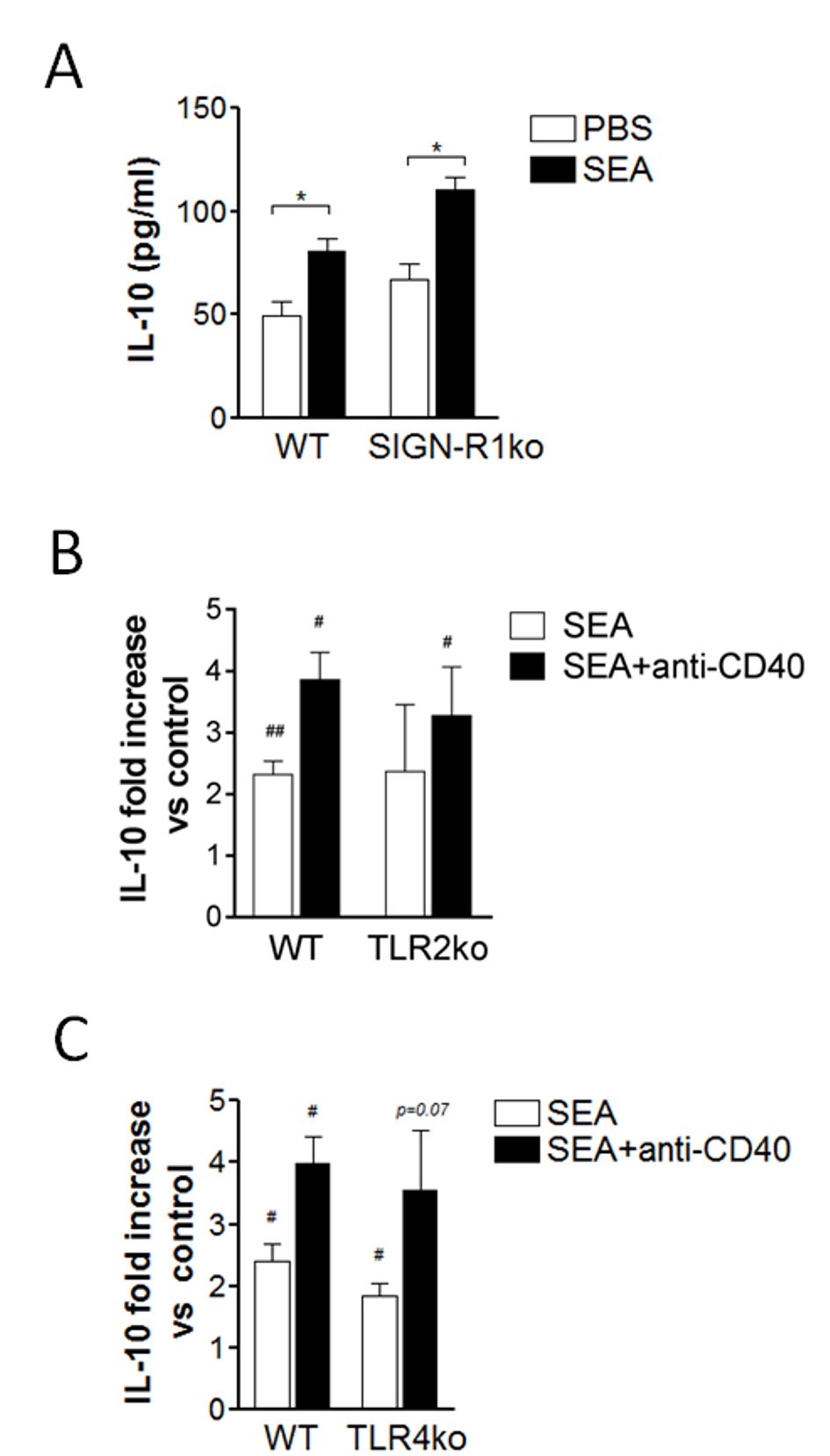

Supplement: S6 Fig — (A) C57BL/6 wild-type or SIGN-R1-deficient mice were treated with 2 doses of SEA (each 100 μg) for 2 weeks or PBS as control. Secreted IL-10 was detected by ELISA after 2 days restimulation of splenic CD19+ B cells with SEA (20 μg/ml). Summary of N = 2–6 mice per group. (B, C) Splenic B cells from naïve wild-type, TLR2-deficient (B) and TLR2-deficient (C) mice were cultured for 3 days with 20 μg/ml SEA or medium as control, with or without addition of anti-CD40 (0.5 μg/ml). IL-10 concentration in culture supernatants is expressed as fold increase versus the medium or anti-CD40 control in a summary of 3 experiments. Significance was tested by one-sample t-test of log-transformed data and is indicated by # p < 0.05, ## p < 0.01. (TIF) [file ppat.1006539.s006.tif]

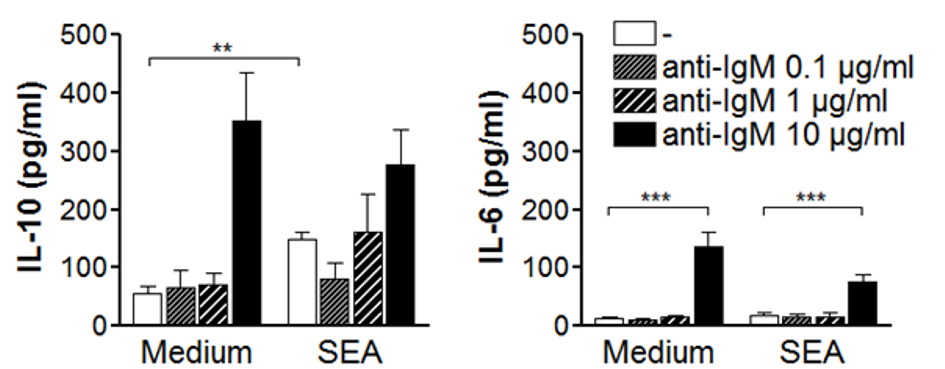

Supplement: S7 Fig — Splenic B cells from naïve mice were cultured with 20 μg/ml SEA or medium as control, with or without addition of anti-IgM Ab in different concentrations. After 3 days of culture, supernatants were analyzed for IL-10 and IL-6 by ELISA. Summary of 3 experiments. Significant differences are indicated with ** p < 0.01 and *** p < 0.001 as tested by Mann-Whitney test. (TIF) [file ppat.1006539.s007.tif]

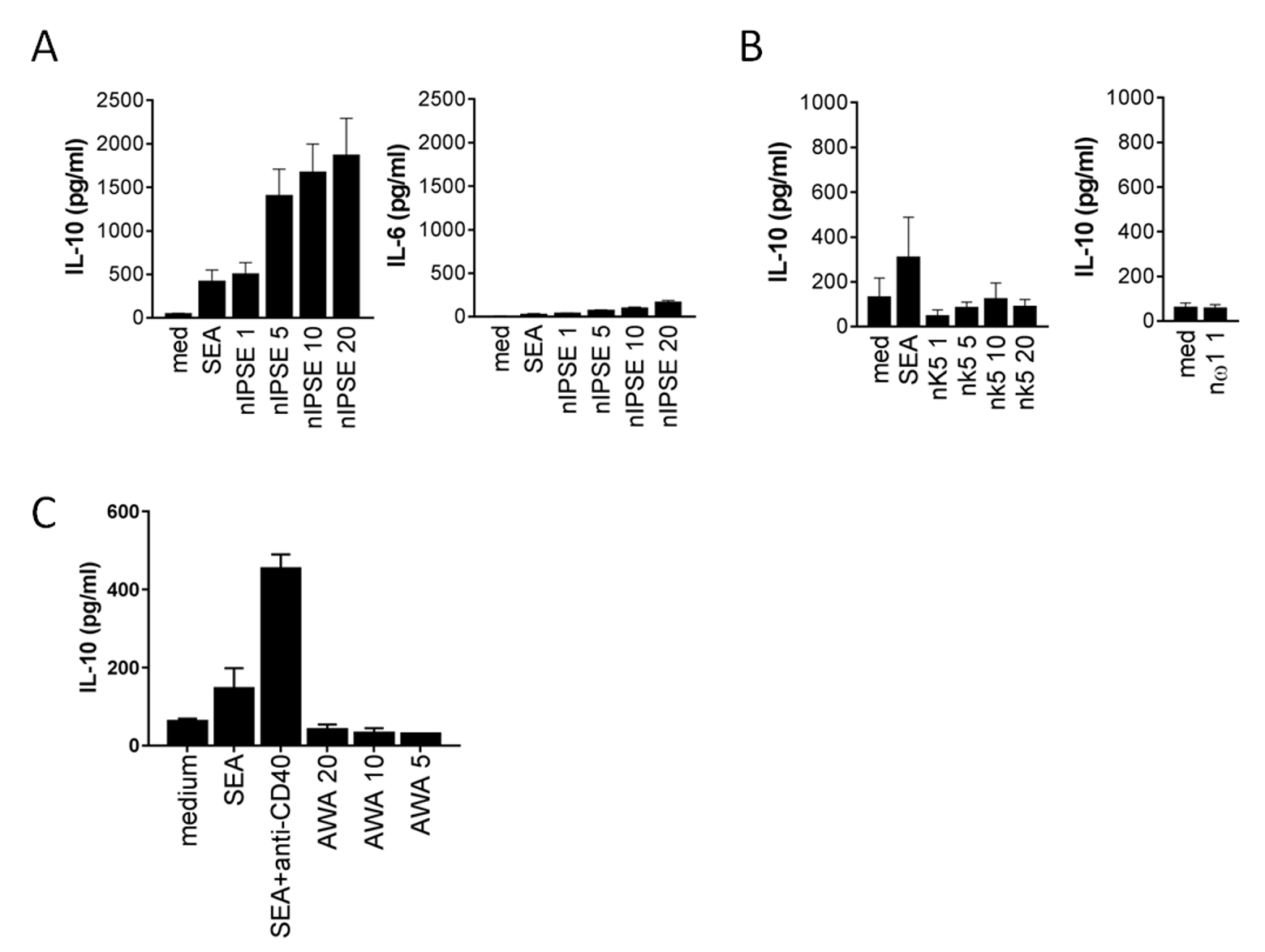

Supplement: S8 Fig — Splenic B cells from naïve mice were cultured for 3 days with different concentrations (indicated by numbers in the x-axis label) of natural (n) IPSE/alpha-1, omega-1 or kappa-5, or medium as negative and 20 μg/ml SEA as positive control. (A) IL-10 and IL-6 concentration in culture supernatants after nIPSE stimulation as measured by ELISA. Average of duplicates from one experiment shown. (B) IL-10 concentration in supernatants of kappa-5 or omega-1 stimulated B cells. Average of duplicates from one experiment shown. (C) Splenic B cells were stimulated in vitro with SEA (20 μg/ml), anti-CD40 (2 μg/ml) or adult worm antigen AWA (5–10–20 μg/ml) for 3 days. IL-10 in culture supernatant as determined by ELISA. Average of duplicate values from one experiment shown. (TIF) [file ppat.1006539.s008.tif]

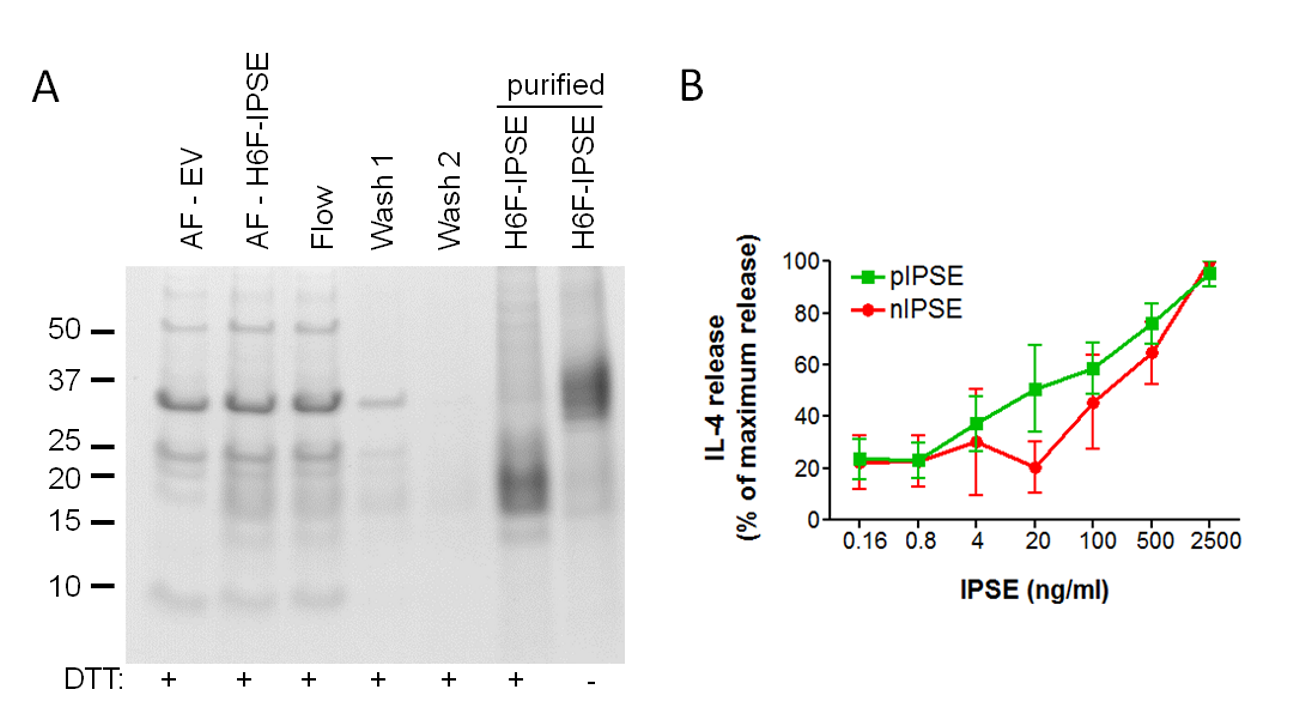

Supplement: S9 Fig — (A) SDS-PAGE and Coomassie blue staining of apoplast fluids (AF) from empty vector (EV), N-terminally tagged H6F-IPSE infiltrated N. benthamiana plants and subsequent small-scale purification of IPSE using Ni-NTA resin and the Äkta Prime purification system. Purified IPSE was analysed under reducing and non-reducing conditions (± DTT). (B) IL-4 release from isolated human basophils during 18h stimulation with pIPSE and nIPSE (0.16–2500 ng/ml), as determined by ELISA. Data are presented as percentage of maximum IL-4 release. Summary of N = 4–6 donors. (TIF) [file ppat.1006539.s009.tif]
